# Evidence of an activity-enhancing conformational shift in *Arabidopsis thaliana* plant cysteine oxidase 4 induced by binding of substrate or substrate-mimics

**DOI:** 10.1016/j.jbc.2025.110770

**Published:** 2025-09-29

**Authors:** Rebecca Latter, Jordi C.J. Hintzen, Laila M.N. Shah, Dona M. Gunawardana, Roberta M. Sher, Mark D. White, Jasmin Mecinović, Justin L.P. Benesch, Emily Flashman

**Affiliations:** 1 Department of Chemistry, University of Oxford, Oxford, UK; 2 Department of Physics, Chemistry and Pharmacy, University of Southern Denmark, Odense, Denmark; 3 Kavli Institute for Nanoscience Discovery, University of Oxford, Oxford, UK; 4 School of Chemistry, University of Sydney, Sydney, New South Wales, Australia; 5 Department of Biology, University of Oxford, Oxford, UK

**Keywords:** enzyme dynamics, Cys sulfinic acid, Cys/Arg N-degron pathway, dioxygenase, peptidomimetics

## Abstract

Plant cysteine oxidases (PCOs) are a family of O_2_-dependent, thiol dioxygenase enzymes that help to coordinate plant responses to flooding by determining the stability of hypoxia-related transcription factors, group VII ethylene response factors (ERF-VIIs). Under normoxia, PCOs use O_2_ to catalyze the oxidation of the Nt-Cys of ERF-VIIs. The resultant N-degron proceeds along the Cys/Arg N-degron pathway for degradation. Conversely, hypoxic conditions such as those experienced during flooding, decrease PCO activity, stabilizing ERF-VIIs which proceed to upregulate hypoxia responsive genes, enabling adaptations to submergence. PCOs are a target for improving plant flood tolerance. Inhibition of PCOs may prepare plants for submergence by promoting upregulation of hypoxia responsive genes. Previous work identified small molecule inhibitors of AtPCO4 and demonstrated that their use as a pretreatment improved seedling tolerance to subsequent anoxia exposure. In this work, the pursuit of a peptide-based inhibition approach led to the development of ERF-VII derived peptidomimetics with modified N termini. Upon testing *in vitro,* the peptidomimetics unexpectedly enhanced, rather than inhibited, AtPCO4 activity. Furthermore, anaerobic preincubation of PCOs with substrate itself was found to induce a similar response. Hydrogen-deuterium exchange mass spectrometry indicated that preincubation with peptidomimetic induces a conformational change in PCO structure. This suggests that substrate binding under anaerobic conditions could promote “conformational priming” of AtPCO4, where flexible regions within or surrounding the active site adopt a conformation that favors enhanced enzymatic activity. These data are the first evidence for dynamic movement of PCO structures and may be of significance for post-hypoxia PCO activity *in planta*.

In both plants and animals, many biochemical pathways require molecular O_2_ and have evolved to proceed optimally under atmospheric levels of O_2_ (normoxia). Plant and animal cells employ the same overarching mechanism to sense when O_2_ availability is reduced (hypoxia) and coordinate adaptive responses. This sensing is achieved by enzymes that regulate the stability of hypoxia-responsive transcription factors in an O_2_ dependent manner (reviewed in (1, 2, 3, 4)). Broadly, adaptive responses include metabolic reprogramming from an aerobic to an anaerobic fermentative system that enables ATP production to continue with reduced O_2_ availability (5, 6, 7). Specific adaptations in animals include improved O_2_ carrying capacity and delivery of blood (8). O_2_ delivery in plants is not facilitated by a dedicated circulatory system but instead relies on diffusion; hypoxic conditions arise when there are barriers to diffusion. Internal barriers to diffusion in certain organs such as the seeds (9) and shoot apical meristems (10) can generate hypoxic niches which are important cues for growth and development while external barriers, such as the soil or submergence under water, result in more widespread hypoxia that, when prolonged, can be detrimental for plant survival (reviewed in (4, 11, 12)). The frequency and duration of flooding events is increasing as a result of climate change (13). Understanding how plants sense and respond to flooding induced hypoxia is important for developing solutions that improve submergence tolerance, particularly in the context of food security.

Hypoxia responses in plants are coordinated by O_2_-sensing enzymes known as the plant cysteine oxidases (PCOs) (14, 15). They regulate the stability of group VII ethylene response factor (ERF-VII) transcription factors, responsible for upregulating hypoxia-responsive genes (14, 16). Forty-nine core hypoxia-responsive genes have been identified and encode proteins including sucrose synthases and ATP-dependent-phosphofructokinases for increased glycolysis, and pyruvate decarboxylases and alcohol dehydrogenase both key enzymes in NAD^+^ regeneration following glycolysis (5, 6, 17). ERF-VIIs undergo N-terminal methionine cleavage by MetAPs, exposing an N-terminal Cys (18, 19, 20). The oxidation state of this N-terminal Cys dictates whether ERF-VIIs are degraded *via* the Cys/Arg N-degron pathway (NDP) (16, 21, 22). O_2_-dependent PCOs regulate the fate-determining step in this pathway (Fig. 1) (14, 15). When sufficient O_2_ is present, PCOs catalyze oxidation of the Nt-Cys to Cys-sulfinic acid (14, 16). This generates a substrate for arginylation by arginyltransferase enzymes and subsequent ubiquitination by PRT6, an E3 ubiquitin ligase (16, 23, 24). Polyubiquitination marks the ERF-VII for degradation *via* the 26S proteosome (23, 25). Under hypoxia, ERF-VII degradation is impeded as the lack of O_2_ prevents PCO activity. This results in ERF-VII stabilization, initiating adaptive responses to hypoxia. Nitric oxide has also been linked to ERF-VII stability, although this step does not appear to relate to PCO activity and the role of NO in the Arg/Cys NDP is yet to be determined (16, 26, 27, 28).

**Figure 1:**
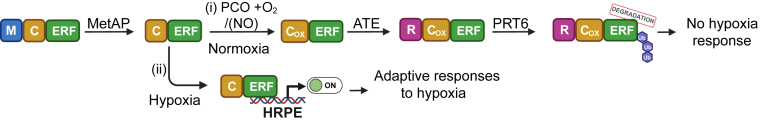
**Oxygen-dependent regulation of ERF-VII stability *via* the Cys/Arg N-degron pathway.** MetAP, methionine aminopeptidase; PCO, plant cysteine oxidase; ATE, arginyl transferase; PRT6, proteolysis6; ERF-VII, group VII ethylene response factor; HRPE, hypoxia response promoter element; single letter code used to denote amino acids.

Crystal structures resolved for three of the five PCOs in *Arabidopsis thaliana* (AtPCO2, 4 and 5) identify them as members of the cupin superfamily of enzymes with their active sites in the core of a jelly roll β-barrel structure, centered on a nonheme Fe^2+^ coordinated by a 3 x His facial triad and 3 x H_2_O (29, 30). This structure is typical of Fe-dependent thiol dioxygenases (TDOs), though comparison with TDOs that exclusively oxidize small molecule substrates suggests the PCOs (and their mammalian homolog, cysteamine dioxygenase (ADO)) have certain structural features that enhance their activity toward N-terminal Cys protein substrates (reviewed in (31)). Seven AtPCO substrates have been confirmed to date; 5 ERF-VII transcription factors (RELATED TO APETALA (RAP) RAP2.2, RAP2.3, RAP2.12, hypoxia responsive ERFs (HRE) HRE1 and HRE2) responsible for the upregulation of hypoxia-responsive genes (6, 14, 15, 21, 22, 32), and two non-ERF-VII proteins, VERNALIZATION2 (VRN2) (33) and LITTLE ZIPPER2 (ZPR2) (10), abundant in hypoxic niches associated with plant development.

Modulation of the Arg/Cys NDP pathway may offer a means to improve plant resilience to submergence by manipulating ERF-VII stability and therefore the expression of hypoxia responsive genes (reviewed in (34)). Permanent ERF-VII stabilization, for example by *ate1ate2* or *prt6* knockout mutations or by modification of the N-terminal Cys of RAP2.12 with a hemagglutinin tag or removal of 13 N-terminal amino acids, results in stunted growth under normoxia and reduced submergence tolerance (22). However, strategies for promoting temporary stabilization of ERF-VIIs, such as overexpressing ERF-VIIs (6, 22), inhibiting the PCOs (35), increasing NO scavenging (36), or reducing *prt6* expression (37), can preadapt plants to survive subsequent hypoxia by promoting the expression of hypoxia-responsive genes. Recently, we reported the first small molecule inhibitors of AtPCO4 and demonstrated their ability to prepare 7-day old *A. thaliana* seedlings in preparation for subsequent anoxia treatment (35).

Here, we explore an emerging area in pharmaceuticals and plant protection, the use of an alternative peptide-based approach for inhibition (reviewed in (38, 39, 40, 41)). Peptide inhibitors can offer greater potency and specificity compared to small molecules as well as an improved safety and toxicology profile toward the environment and nontarget organisms. In addition, they may also inform scaffold-based design of small molecule inhibitors (42). We designed a suite of inhibitory peptides to mimic the sequence of the ERF-VII, RAP2.12_2−15_. These peptidomimetics each feature a Cys-analogue at their N terminus in place of Cys. We predicted that these peptidomimetics would show high specificity for AtPCO4 due to their similarity to an endogenous substrate, resulting in competition with substrate for binding interactions and therefore inhibition of AtPCO4. Surprisingly, *in vitro* assay screenings of peptidomimetics using recombinant AtPCO4 revealed that they enhanced, rather than inhibited, AtPCO4 activity toward a RAP2.12_2—17_ substrate. Upon further investigation our results suggest that AtPCO4 activity enhancement offered by peptidomimetics may arise from a conformational change in the enzyme, an effect that may also be conferred by RAP2_2−17_ itself. The results provide insights into the conformational effects of substrate binding to PCO enzymes and identify a potentially biologically significant mechanism in post submergence recovery of plants.

## Results and discussion

### Peptidomimetics enhance AtPCO4 specific activity toward RAP2.12_2—17_

**Figure 2:**
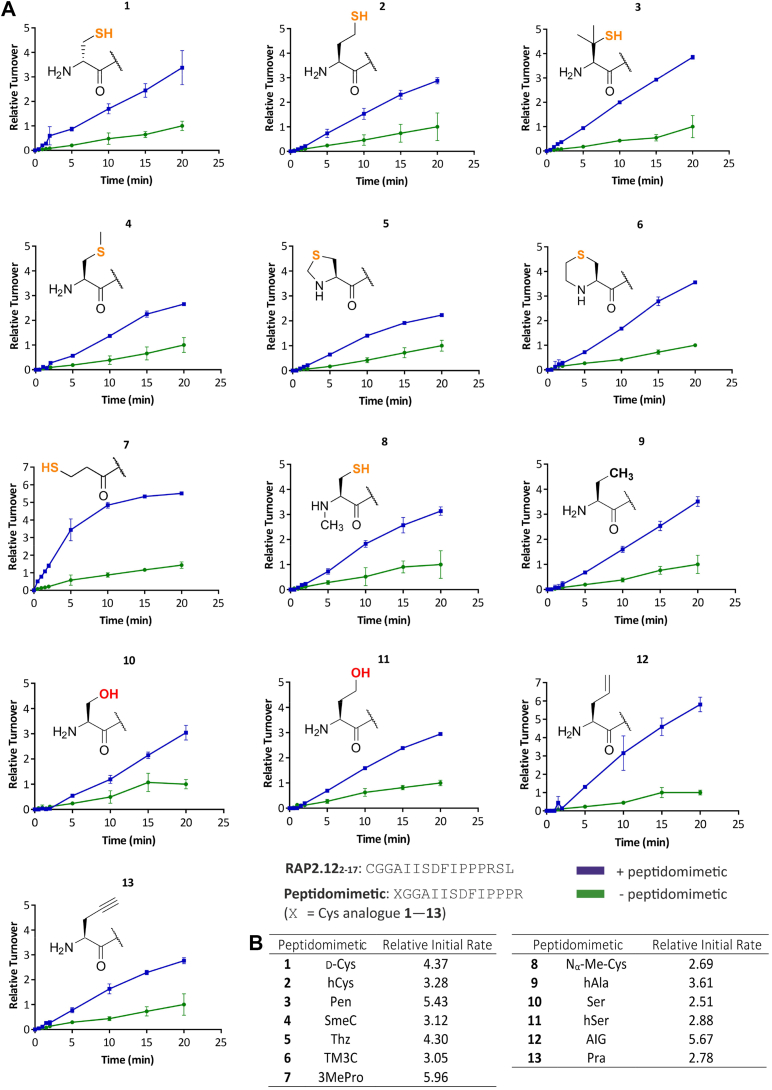
**AtPCO4 activity toward RAP2.12**_**2—17**_**in the presence and absence of peptidomimetics.***A*, *in vitro* time course activity assays showing relative activity of 0.1 μM AtPCO4 toward 20 μM RAP2.12_2—17_ in the absence (*green*) and presence (*blue*) of 200 μM peptidomimetics 1—13 (structures of the peptidomimetic N-terminal Cys-analogue are displayed for each graph). Standard conditions: 5 mM TCEP, 20 μM FeSO_4_, 1 mM ascorbate, 25 °C. Turnover calculated as μmol RAP2.12_2—17_ oxidized per μg of AtPCO4, normalized to the maximum RAP2.12_2—17_ turnover after 20 min in the absence of peptidomimetic (=1). Error bars display S.D (n = 3). *B*, table showing the relative initial rates of RAP2.12_2—17_ turnover, calculated from the rate of reaction between 0 and 5 min in the presence of each peptidomimetic, normalized to the corresponding control condition without peptidomimetic (=1). Within each pair the slopes used to calculate relative rates from 0 to 5 min, differ significantly (determined using Prism 10.3.1). Modified z-score analysis of the set of relative initial scores did not identify any outliers. RAP, RELATED TO APETALA.

Peptidomimetics were designed and synthesized with a 14-mer sequence, XGGAIISDFIPPPR, to represent the Met-cleaved N-terminal sequence of AtRAP2.12 with the reactive Cys substituted for a range of Cys-analogues (X = 1—13, Figs. 2*A* and S1–S13, Table S1). The Cys-analogues explore a range of options for achieving this including (i) inversion of stereochemistry by using d-Cys 1, (ii) extension of the cysteine, as in homocysteine 2, and dimethylation of the β-carbon, as in penicillamine 3 (iii) incorporation of thioether bonds in a linear 4, or cyclized fashion 5—6, (iv) alteration to the N-terminal amino group by removal 7 or methylation 8, (v) substitution of thiol for a methyl or hydroxyl group 9—11 or lower hybridization carbon center 12—13 alternatives. We anticipated that the peptidomimetics would impede substrate binding to AtPCO by competing for points of interaction and therefore inhibit PCO activity.

Before conducting inhibitor screening assays, we first sought to confirm that the peptidomimetics’ N-terminal Cys-analogues did not act as PCO substrates or undergo any modification under assay conditions. Peptidomimetics were therefore incubated with recombinant AtPCO4 for 20 min under aerobic conditions. Subsequent analysis by LC-MS identified no modifications to the peptidomimetics (Fig. S14), confirming that AtPCO4-catalyzed oxidation is specific to N-terminal l-Cys. This enabled us to proceed with screening the peptidomimetics for inhibition toward AtPCO4. In these assays, recombinant AtPCO4 was preincubated in the presence and absence of peptidomimetic for 6 min prior to the introduction of an N-terminal l-Cys substrate peptide, RAP2.12_2—17_ (Fig. 9*A*). A high peptidomimetic-to-RAP2.12_2—17_ ratio (10:1) was set to detect any potential inhibitory properties. The impact of the peptidomimetics on AtPCO4 activity could be assessed by monitoring turnover of RAP2.12_2—17_ over 20 min using LC-MS, as previously described (16, 35). Peaks in the mass spectrum corresponding to 16-mer RAP2.12_2—17_ could be easily distinguished from those of the 14-mer peptidomimetics due to the mass distinction offered by the two additional C-terminal amino acids (Ser-Leu) of the RAP2.12_2—17_ substrate peptide. Contrary to our expectations, the presence of peptidomimetics did not result in inhibition of AtPCO4 activity. Instead, each peptidomimetic tested significantly enhanced the rate of RAP2.12_2—17_ turnover (Fig. 2).

Given that the peptidomimetics from this series were all capable of promoting AtPCO4 activity (Fig. 2*A*), it appeared that the conserved sequence of peptidomimetics is the dominant factor in inducing this response. Furthermore, the sequence similarities between the peptidomimetics and RAP2.12_2—17_ indicate that the response likely arises from substrate-like binding of the peptidomimetics to AtPCO4, but that they are easily displaced by RAP2.12_2—17_ for the reaction to proceed without demonstrating inhibitory effects. The conserved N-terminal Cys of AtPCO4 substrates, likely plays an important role in binding at the AtPCO4 active site and therefore the identities of the Cys-analogues were expected to have a disproportionate influence on the extent of the enhancement compared to the remaining residues of peptidomimetics (15). To assess if the magnitude of the AtPCO4 specific activity enhancement varied depending on the defining N-terminal Cys-analogue of the peptidomimetics, we calculated the initial rates of substrate turnover between 0—5 min time points in the presence of each peptidomimetic, relative to that of the corresponding control reaction in which the peptidomimetic was absent. The initial rate scores (Fig. 2*B*) revealed that AtPCO4 specific activity enhancement by peptidomimetics varied from 2.51-fold, induced by peptidomimetic 10, up to a maximum of 5.96-fold, induced by peptidomimetic 7. Notably, peptidomimetic 7 (the most potent enhancer) features the only Cys-analogue devoid of an amino group which may result in easier displacement by substrate compared to the other peptidomimetics. In contrast, the N-terminal Cys-analogue of peptidomimetic 10 (the weakest enhancer) is a serine and has the greatest similarity to Cys (43), which may lead to it being less promptly displaced by substrate than the other peptidomimetics.

### The substrate-like nature of peptidomimetics leads to priming of AtPCO4 during the preincubation period

**Figure 3:**
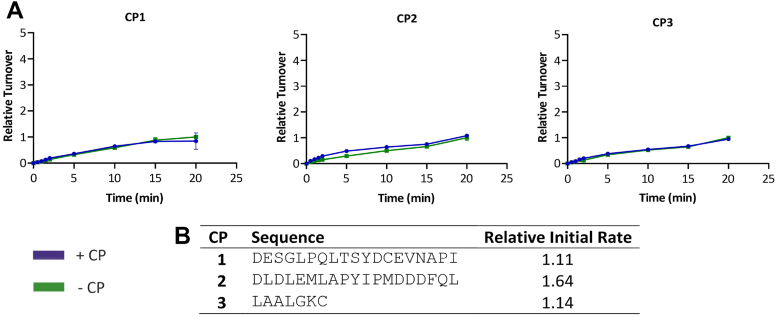
**Testing of control peptides for potential enhancement of AtPCO4 activity.***A*, *in vitro* time course activity assays showing relative activity of 0.1 μM AtPCO4 toward 20 μM RAP2.12_2—17_ in the absence (*green*) and presence (*blue*) of 200 μM CP1—3. All assays conducted under standard conditions: 5 mM TCEP, 20 μM FeSO_4_, 1 mM ascorbate, 25 °C. Turnover is calculated as μmol RAP2.12_2—17_ oxidized per μg of AtPCO4, and turnover values are normalized to the maximum RAP2.12_2—17_ turnover after 20 min in the absence of CP (=1). Error bars display S. D (n = 3). *B*, table showing the relative initial rates of RAP2.12_2−17_ turnover, calculated from the slope of reaction progress between 0 − 5 min in the presence of each CP, normalized to the corresponding control condition without CP (=1). CP, control peptide; RAP, RELATED TO APETALA.

Having observed that the peptidomimetics all induced AtPCO4 activity enhancement, we wanted to confirm that the effect we were observing was due to a specific function of the ERF-VII analogue and rule out a general nonspecific stabilizing effect. It was considered whether AtPCO4 stabilization may arise due to higher concentrations of peptide in the peptidomimetic-containing assay mix compared to the control assay mix. General enzyme stabilization effects can be seen upon the addition of peptide or proteins in enzyme assays, for example bovine serum albumin and β-casein that have been reported to improve enzyme folding and solubility, resulting in increased enzymatic activity (44). We investigated this first by carrying out assays in which the peptidomimetics were substituted for three control peptides (CPs) with peptide sequences unrelated to RAP2.12 and other PCO substrates. AtPCO4 was preincubated either in the presence or absence of each CP for 6 min before introducing RAP2.12_2—17_. AtPCO4 previously exhibited diverging levels of RAP2.12_2—17_ oxidation in the presence and absence of peptidomimetics. However, monitoring the levels of AtPCO4-catalyzed RAP2.12_2—17_ oxidation in the presence and absence of the CPs revealed matching reaction profiles over the 20 min time course (Fig. 3*A*). Calculating the initial rate of reaction between 0 and 5 min in the presence of CPs relative to that in the absence of CP, showed that CP1 and CP3 induced statistically insignificant relative initial rate increases of 1.11- and 1.14-fold, respectively (Fig. 3*B*). Although CP2 induced a statistically significant relative initial rate increase of 1.64-fold, this was much reduced compared to relative initial rate increase generated by the peptidomimetics (for which relative initial rate ≥ 2.51-fold) and, unlike the peptidomimetics, levels of RAP2.12_2—17_ oxidation in CP2-containing reaction and its corresponding control reactions converged after 10 min. These data therefore help to rule out a general stabilizing effect of AtPCO4 in the presence of high peptide concentrations and instead support the effect of binding of the peptidomimetics to AtPCO4 in a substrate-like manner to prime AtPCO4 for increased catalytic activity.

**Figure 4:**
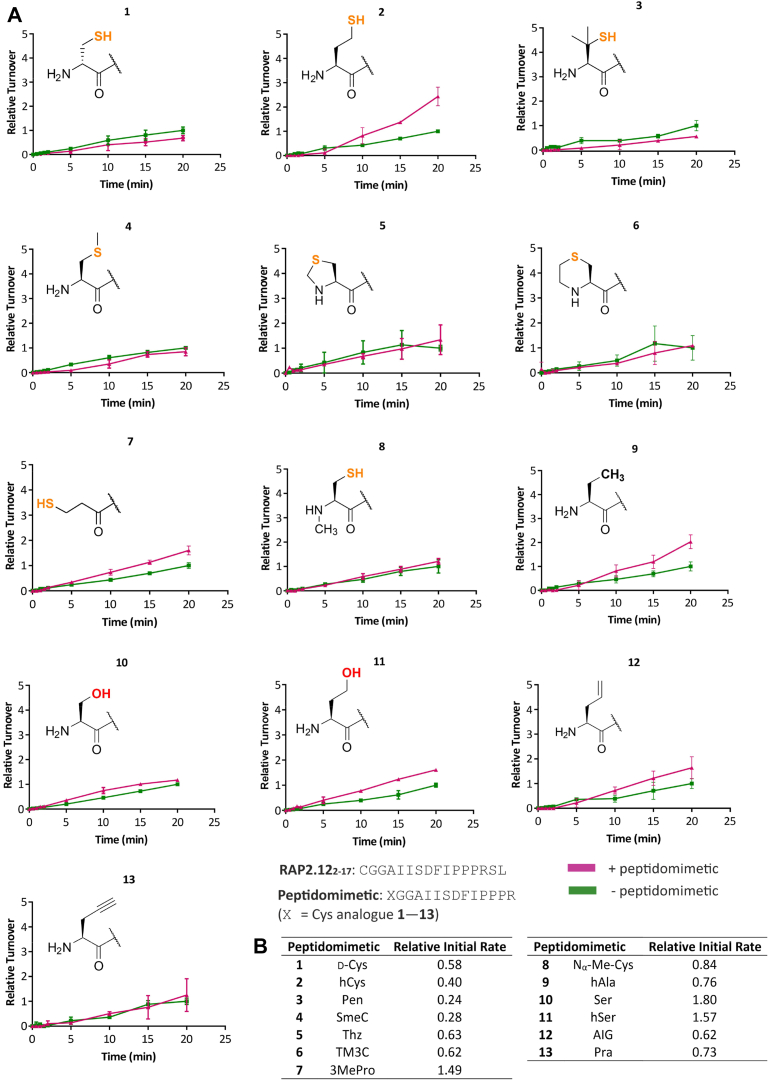
**The effect of removing the peptidomimetic-AtPCO4 preincubation period.***A*, *in vitro* time course activity assays showing relative activity of 0.1 μM AtPCO4 toward 20 μM RAP2.12_2—17_ in the absence (*green*) and presence (*pink*) of 200 μM peptidomimetic, having removed the 6 min AtPCO4-peptdiomimetic preincubation step. All assays conducted under standard conditions: 5 mM TCEP, 20 μM FeSO_4_, 1 mM ascorbate, 25 °C. Turnover is calculated as μmol RAP2.12_2—17_ oxidized per μg of AtPCO4, and turnover values are normalized to the maximum RAP2.12_2—17_ turnover after 20 min in the absence of peptidomimetic (=1). Error bars display S.D (n = 3). *B*, table showing the relative initial rates of RAP2.12_2−17_ turnover, calculated from the slope of reaction progress between 0 − 5 min in the presence of each peptidomimetic, normalized to the corresponding control condition without peptidomimetic (=1). Modified z-score analysis of the relative initial scores identified peptidomimetic 10 as an outlier. RAP, RELATED TO APETALA.

We next hypothesized that peptidomimetic-AtPCO4 binding interactions might be facilitated during their 6 min preincubation. To examine this, we adapted our assay protocol so that AtPCO4 was instead exposed to both the peptidomimetic and substrate simultaneously, thereby preventing preincubation of peptidomimetic with AtPCO4 (Fig. 9*B*). Eleven of the peptidomimetics were tested in this manner and upon doing so, the enhancement effect was found to be significantly reduced (Fig. 4*A*); relative initial rate scores generated over the first 5 min showed 0.24- to 1.80-fold increases in AtPCO4 specific activity in the presence of peptidomimetic (Fig. 4*B*). It could therefore be concluded that preincubation of AtPCO4 with the peptidomimetics is important for promoting their priming effects.

Having observed that the peptidomimetics are unable to competitively inhibit AtPCO4 and validated their unusual ability to enhance AtPCO4 specific activity, it was next considered how peptidomimetic binding to AtPCO4 in a substrate-like manner might translate into enhanced AtPCO4 activity. Given that each of the peptidomimetics enhanced AtPCO4 activity and is structurally similar, we assumed that they operate *via* the same mode of action and therefore investigated potential mechanisms.

We first wanted to rule out the possibility that incubation of AtPCO4 with peptidomimetics could induce an activating modification that has been reported in other TDOs. In the mammalian small molecule TDOs, cysteine dioxygenase and ADO, an activity-enhancing thioether crosslink has been reported, formation of which is induced by high-levels of their small molecule substrates and which is proposed to help regulate responses to substrate fluctuations (29, 45, 46, 47, 48, 49, 50, 51). Although this modification has not been observed in AtPCO4 (29), nor does it have the same need to respond to fluctuating levels of small molecule substrate, we considered it possible that the synthetic peptidomimetics could be inducing a nonnative modification between residues Cys190 and Tyr192 (which correspond to equivalent residues to those in ADO which form a crosslink) (29). Preincubation of a Cys190Ala AtPCO4 variant with peptidomimetic 7 (the most potent peptidomimetic enhancer) for 6 min prior to adding substrate, generated a 5.21-fold increase in the initial rate of RAP2.12_2—17_ oxidation between 0—5 min (Fig. S15). This activity profile matches well with that of WT AtPCO4 (Fig. 2*A*), for which preincubation with peptidomimetic 7 resulted in a 5.96-fold increase in initial rate between 0 and 5 min. This result confirmed that peptidomimetics were not inducing artefactual thioether cross-link formation involving AtPCO4 Cys190.

### Dependency of AtPCO4 priming on RAP2.12_2—17_ and its cosubstrate, O_2_

We next investigated whether the rate-enhancing effect of the peptidomimetics may arise from facilitation of O_2_-binding at the active site ready for oxidation of the N-terminal Cys upon introduction of RAP2.12_2—17_. To test this, we conducted *in vitro* time course assays, analogous to those in Figure 2, but using an anaerobic glove box to eliminate O_2_ during the assay preparation and preincubation period (Fig. 9*C*). This allowed us to identify whether O_2_ is involved in the peptidomimetic-induced priming of AtPCO4 that takes place during this period. Following anaerobic preincubation of AtPCO4 in the presence and absence of peptidomimetic 9, RAP2.12_2—17_ was introduced, and assays were immediately exposed to aerobic conditions to allow for RAP2.12_2—17_ oxidation.

**Figure 5:**
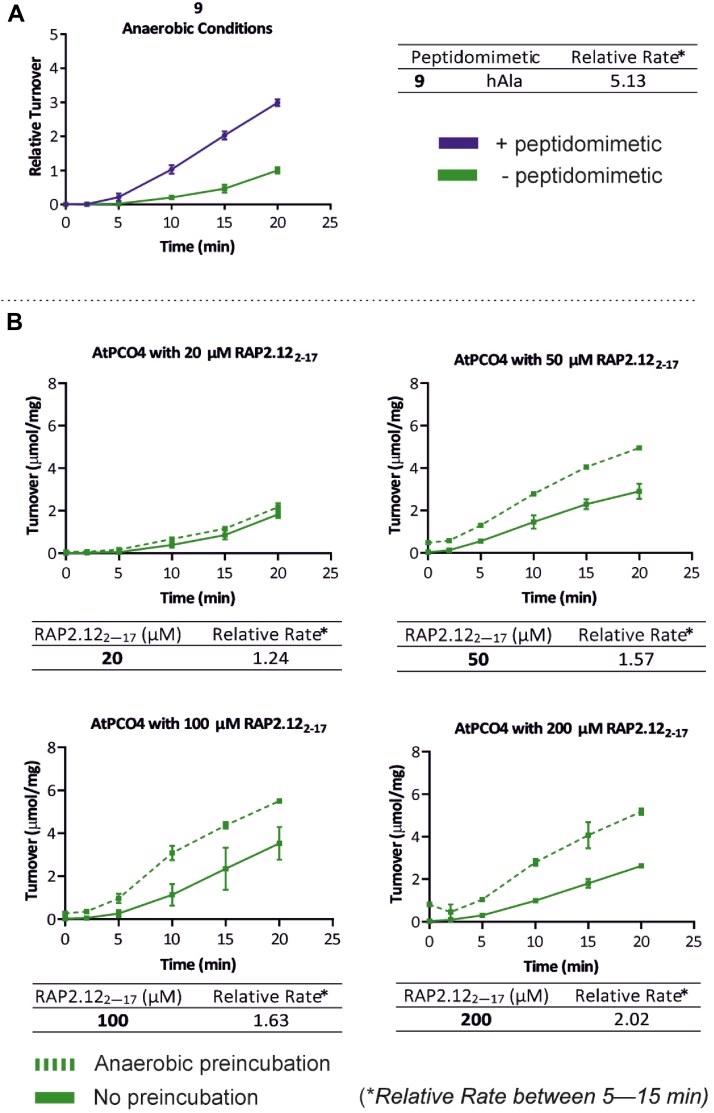
**Investigating the involvement of O_2_ in the peptidomimetic priming response and whether preincubation of substrate can also enhance AtPCO4 activity.***In vitro* time course activity assays showing (*A*) relative turnover of 20 μM RAP2.12_2—17_ by 0.1 μM AtPCO4 in the absence (*green*) and presence (*blue*) of 200 μM peptidomimetic 9, following assay preparation and preincubation under anaerobic conditions. *B*, *in vitro* time course activity assays showing activity of 0.1 μM AtPCO4 toward either 20, 50, 100, or 200 μM RAP2.12_2—17_ after being preincubated in the presence (*green dashed line*) or absence (*green solid line*) of RAP2.12_2—17_ under anaerobic conditions, prior to exposure to aerobic conditions. Standard conditions are 5 mM TCEP, 20 μM FeSO_4_, and 1 mM ascorbate, 25 °C. Turnover is calculated as μmol RAP2.12_2—17_ oxidized per μg of AtPCO4, for (*A*) turnover values are normalized to RAP2.12_2—17_ turnover after 20 min, in the absence of peptidomimetic (=1). Error bars display S. D (n = 3). Relative rate of each reaction is displayed in each accompanying table, calculated from the slope of reaction progress between 5 and 15 min in the presence of peptidomimetic 9, normalized to the corresponding control condition without peptidomimetic (=1) for (*A*), and between 5 and 15 min following anaerobic preincubation with RAP2.12_2−17_, normalized to the corresponding control condition without preincubation (=1) for (*B*). RAP, RELATED TO APETALA.

The results revealed low levels of RAP2.12_2—17_ turnover between 0 and 5 min both with and without peptidomimetic 9 (Fig. 5*A*), which we attribute to a reduced concentration of dissolved O_2_ in the assay following anaerobic preincubation. The rate of turnover then increased as O_2_ diffused into the assay and revealed that once again the preincubation of AtPCO4 with peptidomimetic 9, even under anaerobic conditions, resulted in enhanced specific activity relative to the control reaction in which peptidomimetic was absent. The rate of reaction was calculated using the linear portion of the graph between 5 and 15 min time points, once O_2_ diffusion appears to no longer be a rate limiting factor. A 5.13-fold increase in the reaction rate was generated in the presence of peptidomimetic 9. Although this relative rate value cannot be compared to the relative initial rates calculated for experiments conducted under aerobic conditions, these results nevertheless indicate that peptidomimetic priming of AtPCO4 occurs irrespective of whether O_2_ is present during the peptidomimetic-AtPCO4 preincubation period. It therefore appears that priming does not proceed *via* a mechanism directly mediated by O_2_ preparation at the AtPCO4 active site.

We next wanted to investigate whether anaerobic preincubation of RAP2.12_2—17_ substrate itself with AtPCO4 (*i.e.* without turnover), may similarly be able to enhance AtPCO4 activity. Under anaerobic conditions, AtPCO4 was either preincubated with RAP2.12_2—17_ or in the absence of RAP2.12_2—17_ (control conditions) for 6 min. For the control condition, RAP2.12_2—17_ was prepared under anaerobic conditions and introduced to AtPCO4 immediately before reaction initiation (*i.e.* no preincubation). After 6 min both experiments were exposed to aerobic conditions, initiating the reaction (See Fig. 9*D* for an overview of experimental set up). Crucially, the same concentration of RAP2.12_2—17_ was used for experiments with and without AtPCO4-RAP2.12_2—17_ preincubation. Previous reactions featuring peptidomimetics tested a 10:1 peptidomimetic to RAP2.12_2—17_ ratio, the experimental set up of RAP2.12_2—17_-only experiments means that this could not be replicated. Instead, to ensure that observations were not limited by peptide concentrations, RAP2.12_2—17_-only experiments were repeated for 20, 50, 100 and 200 μM of RAP2.12_2—17_ concentrations.

The results confirm that enhanced AtPCO4 activity can be induced by both substrate and substrate-like peptides as anaerobic preincubation of 50, 100, and 200 μM of RAP2.12_2—17_ with AtPCO4 led to significant increases in substrate turnover upon subsequent exposure to aerobic conditions relative to their respective control assays (Fig. 5*B*). As seen previously for assays preincubated under anaerobic conditions, turnover between 0 and 5 min was limited by O_2_ diffusion into the assay, so relative rates were determined for the reaction between 5 and 15 min. This highlighted that nonreactive preincubation of 20, 50, 100, or 200 μM RAP2.12_2—17_ with AtPCO4 increased enzyme specific activity by 1.24, 1.57, 1.63, and 2.02-fold, respectively with the level of AtPCO4 change being dependent on the concentration of the RAP2.12_2—17_ peptide.

### Peptidomimetic binding mimics substrate binding in the substrate binding channel

**Figure 6:**
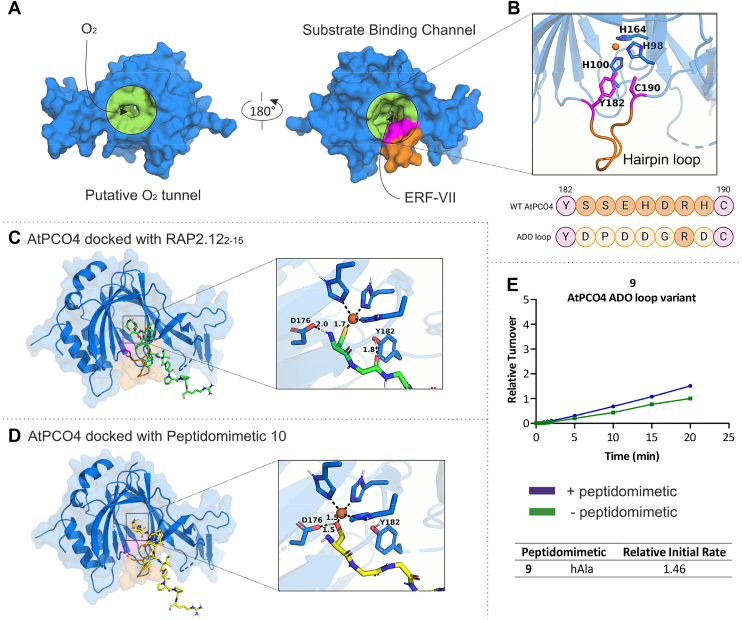
**Docking predictions of RAP2.12**_**2—15**_**and peptidomimetic binding to AtPCO4.***A*, surface representation (*blue*) of AtPCO4 PDB:6S7E (Fe displayed as an *orange sphere*) showing a view of the putative O_2_ entrance tunnel encompassed in a *lime circle* and a 180° rotation presents a view of the substrate binding channel again encompassed in a lime circle but with potential cross-linking residues shown in *pink* and the hairpin loop region in *orange*. *B*, cartoon representation of AtPCO4 displaying the hairpin loop region in *orange* along with potentially cross-linking residues, Cys190 and Tyr182, in *pink*, and 3 x His triad coordinating Fe at the active site. The hairpin loop sequences of ADO and WT AtPCO4 are displayed below with residues represented by *circles*. *C* and *D*, AtPCO4 PDB:6S7E, docked with (C) RAP2.12_2—15_ (*green*, *sticks*) and (D) peptidomimetic 10 (*yellow*, *sticks*). Insets show interactions of the N-terminal residues binding at the active site. Docked structures were generated using the HADDOCK web server and visualized in PyMOL. *E*, *In vitro* time course activity assays showing relative turnover of 20 μM RAP2.12_2—17_ by 0.1 μM AtPCO4 loop variant following preincubation of the enzyme in the absence (*green*) and presence (*blue*) of 200 μM peptidomimetic 9. Standard conditions: 5 mM TCEP, 20 μM FeSO_4_ and 1 mM ascorbate, 25 °C. Turnover is calculated as μmol RAP2.12_2—17_ oxidized per μg of AtPCO4, and turnover values are normalized to RAP2.12_2—17_ turnover after 20 min in the absence of peptidomimetic (=1). Error bars display S. D (n = 3). ADO, cysteamine dioxygenase; RAP, RELATED TO APETALA; PDB, Protein Data Bank.

We next used docking studies to interrogate the potential binding interactions of the peptidomimetics with AtPCO4. The jelly roll β-barrel core seen in crystal structures of AtPCO4 creates a channel through which the active site is accessible *via* two routes. O_2_ is speculated to access the AtPCO4 active site *via* the narrower channel entrance (Fig. 6*A*) (29, 30). Meanwhile, there is evidence to suggest that the wider channel entrance accommodates binding of N-terminal Cys protein substrates, hence it is referred to as the substrate binding channel (Fig. 6*A*) (29). The proposed substrate binding channel in PCOs closely resembles that of ADO; both enzymes have similar active site architectures with high conservation of active site residues and the presence of a flexible hairpin loop region (spanning residues 182—190 in AtPCO4 and residues 212—220 in ADO) (29, 31, 43, 52, 53). Despite a conserved Arg, along with Cys and Tyr residues that bookend the hairpin loop region, the sequence of the hairpin loop region is distinct for PCO and ADO. A high proportion of Asp residues gives rise a greater proportion of negatively charged residues in the hairpin loop region of ADO compared to PCO, allowing speculation that this region has a role in recognition and binding of their different protein substrates (Fig. 6*B*) (29, 52, 53).

To visualize potential interactions of RAP2.12 and the peptidomimetics with AtPCO4, the HADDOCK v2.2 docking platform was able to offer well-informed alternatives to crystal structure data by offering flexible peptide-protein docking (54, 55). The crystal structure of AtPCO4, Protein Data Bank (PDB):6S7E, provided the protein structure required for docking. RAP2.12 is highly disordered and there is currently no crystal structure resolved for this protein; input docking structures for RAP2.12_2—15_ and peptidomimetic 10 were therefore derived from an AlphaFold predicted structure of AtRAP2.12. RAP2.12_2—15_ was used rather than RAP2.12_2—17_ for a better comparison to peptidomimetics. The additional 2 C-terminal amino acids do not appear to affect the ability of the peptidomimetic to enhance specific activity (Fig. S16) (https://alphafold.ebi.ac.uk/entry/Q9SSA8). Peptidomimetic 10 was selected to represent the peptidomimetic series because currently most computational docking methods for peptides and proteins are limited by their ability to only recognize natural amino acids; therefore, all the peptidomimetics aside from peptidomimetic 10 have incompatible N-terminal Cys-analogues.

The HADDOCK docking platform requires the selection of active residues. These are classified as residues experimentally determined to be involved in the interaction between the two molecules; the N-terminal residues of RAP2.12_2—15_ and peptidomimetic 10 were assumed to interact with the active site Fe of AtPCO4, meanwhile site-directed mutagenesis studies have highlighted the importance of active site residues Asp176 and Tyr182 for enzyme activity (29, 30, 43).

RAP2.12_2—15_ and peptidomimetic 10 structures were each docked with AtPCO4 and, perhaps unsurprisingly given their sequence homogeneity, the top docked poses showed a high degree of similarity (Fig. 6, *C* and *D*). Both peptides displayed preferential occupancy of the hypothesized substrate binding channel (matching what has previously been reported for docking of RAP2.12_2—8_ (35)) helping to rule out an allosteric binding mode. Monodentate coordination *via* the N-terminal thiol of RAP2.12_2-15_ or the hydroxyl group of peptidomimetic 10 to the active site Fe appears to be stabilized by additional hydrogen bonding interactions of Asp176 to the N-terminal amino group of RAP2.12_2—15_ (as has been observed in the modeled structure of ADO with a substrate representing peptide, RGS5_2—5_ (53)) or the N-terminal hydroxyl group of peptidomimetic 10. The role of Tyr182 in binding was less well-defined; a hydrogen bonding interaction may form with a backbone amide carboxyl group of RAP2.12_2—15_, but no such interaction was conserved for peptidomimetic 10. Although docking predictions consistently favor monodentate coordination of substrate and substrate-like peptides for ADO and PCO, bidentate binding cannot be ruled out; recent crystal structures of ADO bound to a cyclic peptide inhibitor bearing Nt-Ser substrate analogue motifs revealed coordination to the active site metal (Co^2+^) *via* both the hydroxyl and amino groups of the Nt-Ser. These interactions were supported by hydrogen bonding with Tyr212 and Asp206, respectively (equivalent to Tyr182 and Asp176 in AtPCO4) (43). This ADO-cyclic peptide bound structure also displayed a ∼2 Å shift in the hairpin loop region of ADO (43). Interestingly, RAP2.12_2—15_ and peptidomimetic 10 bound to AtPCO4 in the trajectory of the hairpin loop region that borders the substrate binding channel. The role of this flexible loop region in facilitating enzyme function is not fully understood but its potential involvement in substrate (and peptidomimetic binding) is reinforced by docking results.

### A hairpin loop region in AtPCO4 is involved in peptidomimetic-induced priming

We wanted to assess whether the flexible hairpin loop region, implicated in substrate and peptidomimetic binding by docking experiments, may be involved in the priming mechanism. This region displays high sequence variability among all five AtPCOs, as well as ADO, and this variability may help account for their distinct substrate specificities (29, 52, 53). We replaced the hairpin loop region of AtPCO4 with the analogous loop sequence from HsADO (HsADO residues 212—220, Fig. 6*B*), generating an AtPCO4 loop variant that could then be used to test the significance of the hairpin loop in peptidomimetic priming.

Assays were conducted in which the AtPCO4 loop variant underwent preincubation either in the presence or absence of peptidomimetic 9 before RAP2.12_2—17_ was introduced. Substrate turnover was monitored over a 20 min period (Fig. 6*E*). Although we predicted that this variant may inherently show reduced activity toward RAP2.12_2—17_, we observed that substrate turnover (when peptidomimetic was not present) was comparable to that of WT AtPCO4 (Fig. S17). However, when the AtPCO4 loop variant was preincubated with peptidomimetic 9, its activity was only enhanced by 1.49-fold relative to the control experiment in which the peptidomimetic was absent. Under the same conditions, peptidomimetic 9 had previously enhanced WT AtPCO4 activity by 3.61-fold. We therefore reasoned that substitution of the hairpin loop region limited the ability of the peptidomimetic to enhance enzyme activity, implicating the hairpin loop region in the peptidomimetic priming mode of action.

### Incubation with peptidomimetics may shift AtPCO4 to a more active conformation

We wanted to understand the structure-activity relationship of how the peptidomimetics interact with the hairpin loop region and lead to priming of AtPCO4. We therefore used hydrogen-deuterium exchange mass spectrometry (HDX-MS) to compare structural and dynamic differences between AtPCO4 prior to, and following, preincubation with the peptidomimetics (56). This technique monitors the rate of exchange of labile hydrogens of the amide protein backbone with deuterium from a labeling solution. The rate of exchange is influenced by factors such as solvent accessibility and structural fluctuations, which can result from ligand binding.

AtPCO4 was incubated in the presence and absence of peptidomimetic 9 (1:1000, AtPCO4-to-peptidomimetic 9, representing a concentration of nine known to elicit near maximal AtPCO4 activity enhancement, Fig. S18) for 6 min before samples were diluted in a D_2_O solution for labeling (pD = 7.4). Additional peptidomimetic 9 was not included in the diluting D_2_O solution (as is typical for HDX-MS experiments monitoring protein-ligand binding interactions) to maintain the AtPCO4-to-peptidomimetic 9 ratio and therefore best determine any conformational change that took place during the preincubation period. Samples were quenched after 15 s, 1 min, 5 min, 30 min, or 60 min of labeling and digested using an immobilized pepsin column. The resultant peptides were analyzed by LC-MS to determine their relative deuterium uptake (DU = total deuterium uptake/theoretical maximum uptake). The change in relative deuterium uptake (ΔDU) by AtPCO4 in the presence of peptidomimetic 9 compared to its absence could then be calculated for each time point and was divided by the number of exchangeable amide hydrogens in the peptide to give the relative fractional uptake (ΔRFU).

Positive ΔRFU values indicate protein segments that underwent higher levels of HDX following preincubation with peptidomimetic 9, suggesting increased solvent accessibility and therefore a level of deprotection of these regions. Meanwhile, negative ΔRFU values identify peptide segments that experience reduced HDX following preincubation with peptidomimetic 9 and therefore represent regions of decreased solvent accessibility that have gained a degree of protection. A hybrid significance testing approach identified peptide segments with statistically significant differences in HDX (*p* > 0.01, Fig. S19) (57). ΔRFU values of significant and nonsignificant peptides were averaged, and the results were overlaid on an AlphaFold 2 modeled structure of AtPCO4 that offers resolution of the disordered loop regions that remain undefined in crystal structure representations (Figs. 6 and S19).

**Figure 7:**
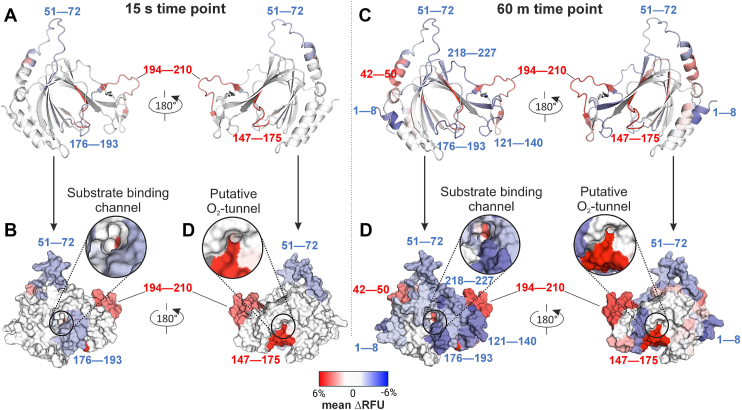
**Visualization of the difference in HDX by AtPCO4 following 6 min preincubation with peptidomimetic 9 at 15 s and 60 min sampling points.** Average ΔRFU values of significant and nonsignificant peptides across AtPCO4 at the 15 s and 60 min sampling time points. Results overlaid on an AlphaFold 2 modeled AtPCO4 structure show increased (*red*), decreased (*blue*), or no significant (*white*) deuterium uptake of AtPCO4 upon preincubation with peptidomimetic 9 relative to the D_2_O-treated AtPCO4-only control. Peptide fragments for affected regions are numbered. *A* and *C*, cartoon representations of AtPCO4. *B* and *D*, surface representations of AtPCO4 with insets of the suspected substrate binding channel and putative O_2_ tunnel. HDX, hydrogen-deuterium exchange; RFU, relative fractional uptake.

Peptidomimetic 9 induced only four regions of statistically significant changes in deuterium uptake that remained consistent across sampling time points (spanning residues 51—72, 147—175, 176—193 and 194—210, Fig. 7, *A* and *B*). Notably, two of these peptide segments, spanning residues 51—72 and 176—193, show increased levels of protection following preincubation with peptidomimetic 9 (Fig. S19). Although a role for the 51—72 peptide is not clear, the 176—193 peptide encompasses the residues 182—190 belonging to the hairpin loop region (Fig. 7, *A* and *C*). We propose that the reduced deuterium uptake in this region may arise from peptidomimetic 9 binding to, and thereby protecting, this region from HDX. Increased protection at the site of the substrate binding channel is clearly displayed in the surface representations of AtPCO4 in Figure 7, *B* and *D* and aligns with docking trajectory predictions for RAP2.12 seen previously in Figure 6, *C* and *D*.

The remaining two regions with consistent statistically significant changes in deuterium uptake across sampling time points, revealed increased levels of deprotection following preincubation with peptidomimetic 9 (Fig. S19). Interestingly, these peptide segments, span residues 147—175 and 194—210. Both regions correspond to flexible loop regions on either side of the hairpin loop region (encompassed by peptide segment 176—193) (Fig. 7, *A* and *C*). We hypothesize that an interaction of peptidomimetic 9 with the hairpin loop region may initiate the flexible regions either side of it to undergo a conformational shift, priming AtPCO4. Although a role for the peptide spanning 194—210 is yet to be defined, predicting a role for the peptide spanning 147—175 is more straightforward as this loop forms a tunnel that is proposed to allow O_2_ to enter the active site (52). The proximity of this deprotected region to the putative O_2_-tunnel can be clearly observed in red on the surface representation of AtPCO4 in Figure 7, *B* and *D*. A conformational change in this region could have implications for O_2_ access to the active site *via* the O_2_-tunnel and could contribute to the increased specific activity observed for AtPCO4 following peptidomimetic preincubation.

HDX-MS samples taken after 60 min resulted in the largest number of statistically significant changes in deuterium uptake (Fig. 7, *C* and *D* and S19). In addition to the regions reported above, AtPCO4 regions spanning 1—8, 121—140, and 218—227 also displayed a reduction in deuterium uptake. The AlphaFold 2 representation of AtPCO4 suggests these regions form partially or completely disordered loops; a gain in secondary structure or other intramolecular interactions may account for their increased protection from deuterium exchange (Fig. 7, *A* and *C*).

Overall, results from HDX-MS experiments help rationalize how structural changes to AtPCO4 in the presence of the peptidomimetics might correlate with increases in AtPCO4 specific activity that are observed upon the addition of substrate.

## Conclusion

The initial aim of this work was to identify peptide inhibitors of PCOs in order to explore a potential route to prepare plants for submergence tolerance. Peptidomimetic inhibitor candidates were developed with sequences based on the AtPCO substrate, RAP2.12_2—15_, featuring Cys-analogues in place of the N-terminal Cys. It was anticipated that their substrate similarity may offer highly specific inhibition of PCOs, thereby minimizing the possibility of off-target consequences. After first establishing that AtPCO4 was unable to modify the N-terminal Cys analogues of the peptidomimetics, we were surprised to find that rather than inhibiting the reaction between AtPCO4 and RAP2.12_2—17_, the peptidomimetics enhanced AtPCO4’s catalytic activity toward RAP2.12_2—17_ by 2.51- to 5.96-fold (under the conditions of this study).

**Figure 8:**
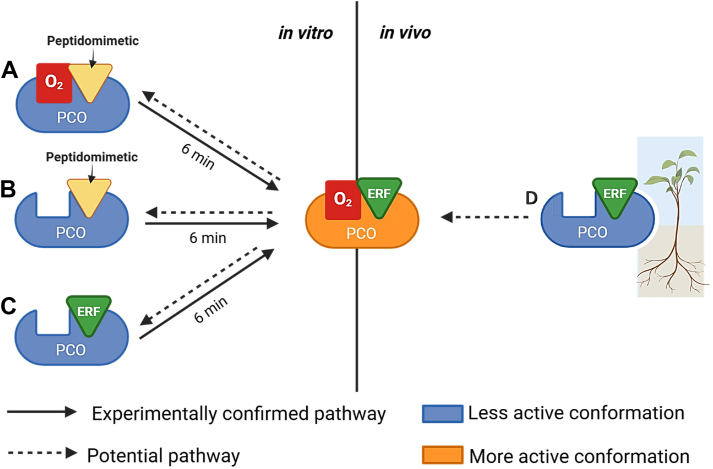
**Overview of the criteria required to enhance AtPCO4 activity.***In vitro*, a 6 min preincubation of peptidomimetics irrespective of the presence of O_2_ (*A* and *B*) or substrate under anaerobic conditions (*C*), primes AtPCO4 for increased specific activity. This may be caused by a shift from a less active conformation (*blue*) to a more active conformation (*orange*). An analogous scenario might occur *in vivo* whereby plants experiencing submergence induced hypoxia are effectively preincubated with ERF-VII substrates (*D*). ERF-VII, group VII ethylene response factor

The enhanced activity was found to be specific to the substrate-like nature of the peptidomimetics and promoted by a period of preincubation (aerobic or anaerobic) prior to substrate introduction (Fig. 8, *A* and *B*). The effect was then found to extend to substrate itself when preincubated under anaerobic conditions (Fig. 8*C*). The rate enhancing effect was found to be coordinated by nonreactive, noncovalent binding of either substrate or the peptidomimetics to a hairpin loop region (encompassed by residues 176—193) near the active site of AtPCO4. This is suspected to prime AtPCO4 by triggering a shift to a more active conformation that might facilitate O_2_ delivery to the active site.

The ability of substrate and substrate-like peptides to induce activity enhancing conformational changes for AtPCO4 may result from positive kinetic cooperativity (58). Cooperativity occurs when prior binding of a ligand alters the affinity of a subsequent binding event. For monomeric, single-ligand binding site enzymes cooperativity is often linked to conformational changes that occur at a rate slower than substrate turnover (58). We have previously reported observations indicating cooperativity for AtPCO4 when determining its binding constant with RAP2.12_2—15_, finding that plots of tryptophan fluorescence quenching against RAP2.12_2—15_ concentrations resulted in a sigmoidal Hill slope (59). Furthermore, this study used nonreactive binding conditions of AtPCO4 with RAP2.12_2—15_, (achieved by using Ni^2+^-substituted AtPCO4 and preparing samples under anaerobic conditions) which were reflective of the nonreactive preincubation conditions we reported as a prerequisite for inducing enhanced AtPCO4 activity.

It has been suggested that positive cooperativity effects may serve a regulatory function in modulating enzymatic responses to changing substrate levels. The best studied example of this for a monomeric enzyme is that of human glucokinase (60). It plays a crucial physiological role as a glucose sensor and its positive cooperativity upon nonallosteric association with glucose drives a conformational change, clearly observed by X-ray crystallography, and resulting in increased responsiveness to biologically relevant blood glucose concentrations (58, 61). Given the role of PCO in metabolic regulation, the positive cooperativity we observed for AtPCO4 might be of biological significance *in planta* (15). Under hypoxia, plants experience reduced O_2_ levels that lead to inhibition of PCOs and therefore an accumulation of ERF-VIIs (14). This scenario emulates the preincubation conditions of our *in vitro* experiments, offering an opportunity for nonreactive binding of ERF-VIIs to PCO. This may drive PCO to shift to a more active conformation that accelerates ERF-VII degradation upon reoxygenation, helping to rapidly restore optimal metabolic functioning (Fig. 8*D*), albeit PCO activity can also be affected by other cellular signals that accumulate during hypoxia (22, 62).

In this study, we focused on AtPCO4 (the most catalytically active of *A. thaliana*’s five PCO paralogs) (15) and RAP2.12-representing peptides (one of the most prominent activators of hypoxia-responsive genes of *A. thaliana*’s 5 ERF-VIIs) (22, 63, 64). Further experiments are required to determine if our observations of positive cooperativity are more broadly applicable to PCOs and ERF-VIIs across plant species both *in vitro* and *in vivo* (12, 65, 66). There are limited methods for observing positive cooperativity and correlating this with physiological importance *in vivo* but *4pco* mutant Arabidopsis plants (lacking *pco1*, *2*, *4*, and *5*) might offer scope for doing so. Complementing *4pco* plants with functional TDOs allows for assessment of their activity by monitoring their ability to reduce developmental defects and restore O_2_-dependent regulation of ERF-VIIs (25, 34). Having established that the hairpin loop region plays an important role in facilitating a conformational shift *in vitro*, a comparison of *4pco* plants complemented with either WT AtPCO4 or the AtPCO4 ADO loop variant could verify the significance of the hairpin loop region *in planta*. This may provide an indication of the extent to which positive cooperativity (*i.e.* AtPCO4 priming by ERF-VIIs under hypoxia) impacts plant recovery following hypoxia treatment.

The ability of peptidomimetic and substrate to enhance AtPCO4 activity increases the rate of RAP2.12_2—17_ oxidation under the conditions tested. *In vivo* such an effect would encourage ERF-VII degradation by enabling progression along the Cys/Arg N-degron pathway. Although the peptidomimetics are not hypothesized to provide any agrochemical benefit, our unusual observations do provide novel insights into PCO enzyme dynamics. In addition, the structural and functional insights reported here will help guide the targeted engineering of the PCO structure to modulate their O_2_ sensitivity and/or substrate specificity for improved plant flood tolerance (34).

## Experimental procedures

### Synthesis of peptidomimetics

RAP2.12_2—17_ were purchased from GL Biochem. All peptidomimetics were chain assembled on Rink amide resin using microwave assisted SPPS on a Liberty Blue peptide synthesizer (CEM corporation) with the equivalent ratio of [5]:[5]:[7.5] of [Fmoc-protected amino acid]:[DIC]:[Oxyma Pure] at 50 °C for 10 min. The final amino acid was coupled by manual SPPS with the equivalent ratio of [2]:[2]:[3.5] of [Fmoc-protected amino acid]:[HATU]:[DIPEA] overnight. Full conversion was checked by Kaiser test. Peptides were cleaved from resin using 0.5% TIPS and 0.5% H_2_O in TFA, the d-cysteine, homocysteine, penicillamine, thiazolidine, and thiomorpholine containing peptides with 0.5% TIPS, 0.5% H_2_O, and 0.5% EDT in TFA, and the *S*-methyl cysteine containing peptide with 0.5% TIPS, 0.5% H_2_O and 10% dimethyl sulfide in TFA. Following TFA removal, the resultant residue was suspended in cold Et_2_O and centrifuged for 5 min 5804R centrifuge (Eppendorf). The white to yellow precipitate was washed 2 x cold Et_2_O and centrifuged again before being dissolved in a mixture of acetonitrile (ACN) in water and purified using preparative reverse-phase HPLC (RP-HPLC) using a gradient of buffer A and buffer B from 20% B to 70% over 40 min at 4 ml min^−1^ using a Gemini 10 μm NX-C18 110 Å LC column (Phenomenex). Analytical RP-HPLC was carried out on a Gemini 5 μm C18 110 Å LC column (Phenomenex) at a flow rate of 1 ml min^−1^. Analytical injections were monitored at 215 nm.

### *In vitro* activity assays

#### Peptidomimetic and CP screening assays (aerobic conditions)

Recombinant AtPCO4 was expressed using BL21(DE3) *Escherichia coli* cells containing the plasmid pET-28a(+)-His-THROMBIN-AtPCO4, and purified using Ni^2+^-affinity and size exclusion chromatography as described in (59). WT AtPCO4 along with AtPCO4 Cys190Ala and AtPCO4 ADO loop variants were purified using this method. WT AtPCO4 reactivity toward the peptidomimetics was assessed by incubating (0.1 μM) recombinant enzyme with (200 μM) peptidomimetic in assay buffer (assay buffer: 50 mM Bis-tris propane, 40 mM NaCl, pH 8) supplemented with 20 μM FeSO_4_, 1 mM ascorbate, 5 mM TCEP, at 25 °C for 20 min prior to quenching in 5% (v/v) formic acid.

**Figure 9:**
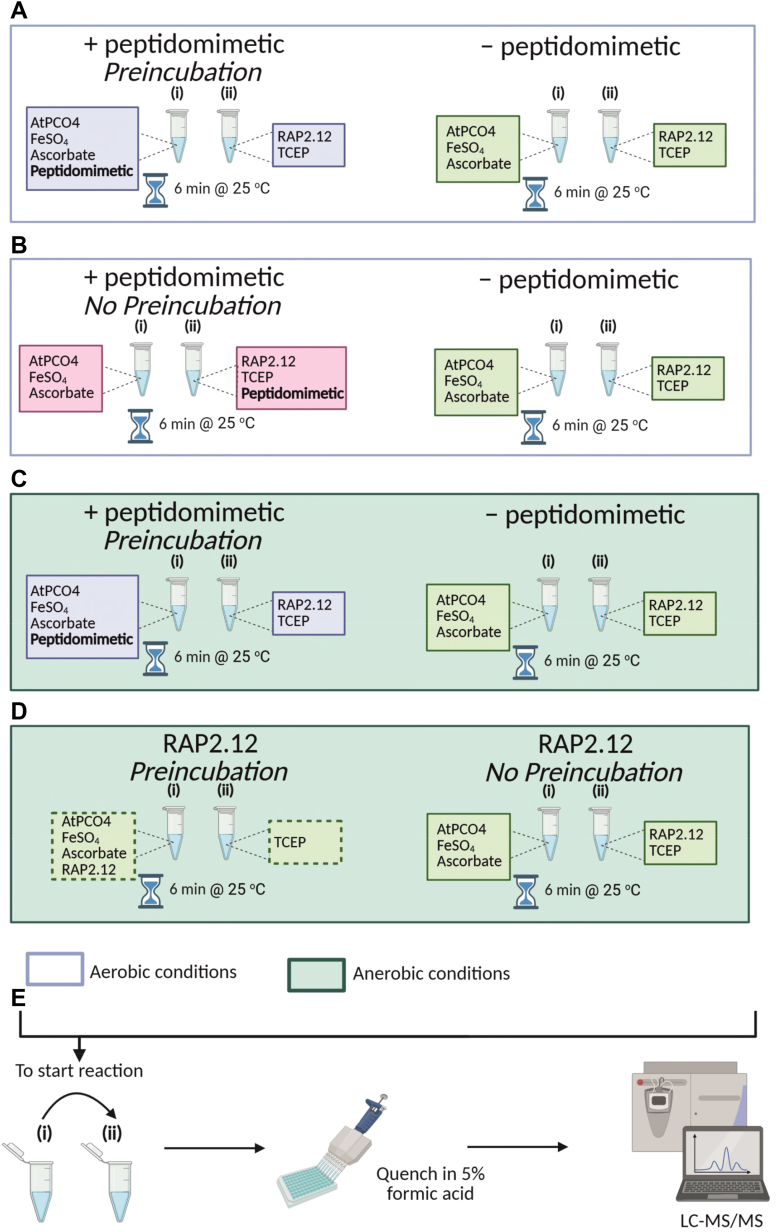
**Overview of assay protocols.***A*, AtPCO4 preincubated in the presence and absence of peptidomimetic; *B*, observing AtPCO4 activity in the presence and absence of peptidomimetic, without peptidomimetic being preincubated with AtPCO4 prior to the reaction; *C*, AtPCO4 preincubated in the presence and absence of peptidomimetic under anaerobic conditions; *D*, AtPCO4 preincubated in the presence and absence of RAP2.12 under anaerobic conditions *E*, following preparation and preincubation of assay components, equal volumes of mixtures (i) and (ii) were combined to achieve the desired reaction concentrations. Reactions were initiated when AtPCO4 and RAP2.12 were present in the same solution under aerobic conditions. Samples were taken at selected time points and quenched in 5% (v/v) formic acid before being analyzed by LC-MS. RAP, RELATED TO APETALA.

To determine AtPCO4 catalytic activity toward RAP2.12_2—17_ in the presence and absence of peptidomimetic (Fig. 9*A*) or CP, mixtures of (i) recombinant enzyme with either 0 or 400 μM peptidomimetic/CP in assay buffer supplemented with FeSO_4_ and ascorbate and (ii) RAP2.12_2—17_, in assay buffer supplemented with TCEP, were incubated separately at 25 °C for 6 min. Equal volumes of mixtures (i) and (ii) were then combined achieving the desired reaction concentrations (0.1 μM recombinant enzyme, 20 μM RAP2.12_2—17_, 0 or 200 μM peptidomimetic/CP, 20 μM FeSO_4_, 1 mM ascorbate, and 5 mM TCEP) and initiating the reaction. Reaction samples taken at 0 s, 30 s, 60 s, 90 s, 2 min, 5 min, 10 min, 15 min, and 20 min intervals were quenched in 5% (v/v) formic acid.

To test the significance of the peptidomimetic-AtPCO4 preincubation step, the method above was modified so that either 0 or 400 μM peptidomimetic was instead in mixture (ii) (Fig. 9*B*). Mixtures were again incubated separately at 25 °C for 6 min before being combined in equal volumes, initiating the reaction. Samples were taken and quenched in 5% (v/v) formic acid.

#### Peptidomimetic screening assays (anaerobic conditions)

In experiments analogous to the above the composition of mixtures (i) and (ii) remained the same however they were prepared and preincubated in an anaerobic glove box for 6 min (Fig. 9*C*). Samples were taken from each mixture and quenched in 5% (v/v) formic acid before combining equal volumes of mixtures (i) and (ii) to achieve the desired reaction concentrations. The assay plate was immediately removed from the anaerobic glove box, exposure to aerobic conditions initiated the reaction and samples were taken at 0 s, 2 min, 5 min, 10 min, 15 min, and 20 min intervals and quenched in 5% (v/v) formic acid.

#### Substrate only assays (anaerobic conditions)

In an anaerobic glove box 40, 100, 200, and 400 μM RAP2.12_2—17_ was either added to mixture (i) containing recombinant enzyme in assay buffer supplemented with FeSO_4_ and ascorbate or mixture (the preincubation condition) or a mixture (ii) containing only assay buffer supplemented with TCEP (the no preincubation condition) (Fig. 9*D*). Each mixture was incubated separately for 6 min under anaerobic conditions before equal volumes of mixtures (i) and (ii) were combined achieving the desired reaction concentrations (0.1 μM recombinant enzyme, 20, 50, 100 or 200 μM RAP2.12_2—17_, 20 μM FeSO_4_, 1 mM ascorbate, and 5 mM TCEP). The assay plate was immediately removed to aerobic conditions, initiating the reaction. Samples were taken at 0 s, 2 min, 5 min, 10 min, 15 min, and 20 min and quenched in 5% (v/v) formic acid.

#### AC_50_ determination

The AC_50_ for peptidomimetic 9 was determined by preincubating peptidomimetic 9 with (i) recombinant enzyme in assay buffer supplemented with FeSO_4_ and ascorbate, at 25 °C for 6 min, under aerobic conditions before combining with an equal volume of mixture (ii) containing RAP2.12_2—17_, in assay buffer supplemented with TCEP (incubated separately at 25 °C for 6 min) to achieve desired reaction concentrations of 0.1 μM recombinant enzyme, 20 μM RAP2.12_2—17_, 0—500 μM peptidomimetic 9, 20 μM FeSO_4_, 1 mM ascorbate, 5 mM TCEP. Nine different peptidomimetic 9 concentrations, ranging from 1—500 μM were tested. Samples were taken after 5 min, quenched in 5% (v/v) formic acid and analyzed by LC-MS.

### Assay analysis by mass spectrometry

All reactions were conducted in triplicate and oxidation of peptides in quenched samples was determined using one of the following mass spectrometry techniques described previously (67): (i) a RapidFire RF 365 high-throughput sampling robot (Agilent) coupled to a 6550 Accurate-Mass Quadrupole Time of Flight (QTOF) mass spectrometer (Agilent) operated in positive ionization mode. Spectra were assessed manually in MassHunter Qualitative Analysis B.07.00 software (Agilent) to ensure the correct ion was chosen for peptide oxidation quantification. RapidFire Integrator.exe was used to integrate areas of product and substrate ions (ii) an Acquity Ultra-high performance liquid chromatography system (Waters) coupled to a Xevo G2-XS Q-ToF mass spectrometer (Waters) operated in positive electrospray mode. Instrument parameters, data acquisition and data processing were controlled by MassLynx V4.1 software (Waters). Source parameters were adjusted to maximize sensitivity and minimize fragmentation. Samples were injected on to a Chromolith Performance RP-18e 100-2 mm HPLC column (Merck) at 40 °C and followed and elution gradient of 95% A/5% B 0—1 min; 5% A/95% B 1—4 min; 95% A/5% B 4—6 min ((A) deionized water supplement with 0.1% (v/v) formic acid (B) ACN), at a flow rate 0.3 ml min^−1^. TOF mass scan range 50.00—2500.00 Da. Spectra were assessed manually in MassLynx V4.1 software (Waters) to ensure the correct ion was chosen for peptide oxidation quantification. MassLynx V4.1 software (Waters) was used to integrate areas of product and substrate ions.

Integration values obtained by MS were subsequently used to quantify reaction turnover (μmol of substrate oxidized per mg of enzyme), and graphs were generated using Prism 10 (GraphPad).

### HADDOCK docking v2.2

AtPCO4 (PDB code: 6S7E, resolution 1.82 Å) was retrieved from RCSB Protein Data Bank (https://www.rcsb.org/) and prepared using the PDB-tools-webserver, generating a.pdb file with single occupancy side-chain conformations. Water molecules were removed, and a 2+ charge was applied to the iron (heteroatom, HETATM) by editing the.pdb text file.

To generate substrate peptide and peptidomimetic files for docking, an AlphaFold predicted structural model of RAP2.12 (https://alphafold.ebi.ac.uk/entry/Q9SSA8) was modified to remove the N-terminal methionine and display only 14 N-terminal amino acids. Editing the.pdb text file for RAP2.12_2—15_, the N-terminal CYS was modified to CYF, removing the hydrogen from the thiol group to improve coordination with Fe^2+^ (known interaction). To build peptidomimetic 10, the RAP2.12 structure file was used as a base and the N-terminal thiol of cysteine was substituted for the hydroxy group to represent a serine residue.

Prepared protein.pdb and peptide.pdb structures were uploaded to the HADDOCK webserver v2.2 (54, 55). Active residues (residues of central importance to docking interactions, known to have roles in substrate binding) selected were D176, Y182, and Fe^2+^ for AtPCO4, and the N-terminal residue for substrate peptide and peptidomimetic 10. The docking experiments were run using default input parameters and submitted structures underwent (i) randomization of orientations and rigid-body minimization (it0) generating 1000 models which were then refined by (ii) semiflexible simulated annealing in torsion angle space (it1) and (iii) refinement in Cartesian space with explicit solvent (water). The final docked models are clustered based on similarities, root mean square deviation (RMSD) calculations determine their deviation from the top ranked model and an assigned z-score indicates how many standard deviations from the average the cluster is located; .pdb files of docked results were visualized in PyMOL (Schrodinger).

### HDX-MS experiments

Recombinant AtPCO4 was expressed using BL21(DE3) *E. coli* cells containing the plasmid pET-28a(+)-His-TEV-AtPCO4 and purified as described above and in (59) but with the addition of a TEV protease cleavage step to remove the N-terminal His_6_ tag prior to size exclusion chromatography (cleavage of the His_6_ tag and use of a codon optimized sequence promoted molecular weight homogeneity of peptide fragments).

AtPCO4 was either preincubated in the presence (1:1000 AtPCO4-to-peptidomimetic 9) or absence of peptidomimetic 9 for 6 min before 5 μl of each sample was diluted in 55 μl deuterated buffer (5 mM potassium phosphate in D_2_O, pD = 7.4) at room temperature to achieve 15 μM AtPCO4 ± 15 mM peptidomimetic 9 and a labeling solution of ∼92% D_2_O. Samples were then incubated for 15 s, 60 s, 5 min, 30 min, and 60 min and then quenched with an ice-cold H_2_O buffer (50 mM potassium phosphate, pH = 1.9) of equal volume. Control (0s) experiments were performed by adding 55 μl of a nondeuterated equilibrium buffer (5 mM potassium phosphate in H_2_O, pH = 7.4) to dilute a recombinant AtPCO4 sample to 15 μM, this was immediately followed by quenching. The pH of the quenched solution was ∼2.5 at 0 °C. This was injected into an on-line HDX manager (Trajan Scientific and Medical) set to 0 °C. The sample was injected onto a 50 μl sample loop before passing over a protease column (packed in house (2 mm × 2 cm) with immobilized pepsin (Thermo Fisher Scientific), at 20 °C using an isocratic H_2_O 0.1% (v/v) formic acid solution at a flow rate of 200 μl/min. Peptide products were collected on a trapping column (VanGuard ACQUITY C18 BEH 2.1 × 5 mm; Waters), at 0 °C. After 3 min collection and desalting, peptides were eluted onto an analytical column for separation using a reverse-phase gradient with a flow rate of 80 μl/min. The elution profile using a H_2_O (A) ACN (B) 0.1% (v/v) formic acid gradient was as follows: 1—7 min, 92% (A) to 65% (A); 7—8 min, 5% (A) to 5% (A); 8—10 min hold at 5% (A). This was followed by a saw tooth gradient between 95% and 5% (A) for 10 min to backflush the columns between proteins runs. This was then followed by a re-equilibration period of 2 min at 92% (A). The analytical flow rate was 40 μl/min, and was electrosprayed into a Cyclic IMS QTOF MS (Waters) for mass analysis. Sample handling was automated using a robotic liquid handling HDX3 system (Trajan Scientific and Medical) to ensure reproducible timings.

Instrument conditions were as follows: capillary 3.0 kV, sample cone 30 V, source offset 30 V. The source temperature was set to 80 °C, the desolvation temperature was 150 °C and the desolvation gas flow was 600 L/h, nebulizer gas 6.5 L/h, trap collisional energy 6 V, transfer collisional energy 4 V. Spectra were acquired between 50—2000 m/z. LeuEnk was used as an internal calibrant and acquired every 30 s 0 s MSE data were acquired for peptide mapping where the transfer collisional energy is ramp from 20 V—30 V, and sample cone is overridden to 50 V.

Peptides were identified from MS/MS data collected from the control runs and processed using Protein Lynx Global Server 3.0 (Waters). This was then used to create a database on DynamX 3.0 (Waters) to assign spectra from the labeling runs using the following parameters; minimum sequence length 5, maximum sequence length 25, minimum fragmentation products per amino acid 0.2, maximum MH + Error (ppm) 5 and file threshold 3. The automated peak analysis was then verified manually to remove any noisy, overlapping or ambiguous charge states and peptides. The change in deuterium uptake was then calculated by comparing the shift in m/z over time to the nondeuterated control. These data were then exported and statistically analyzed using Deuteros 2.0 (Andy Lau) and visualized using PyMOL (Schrodinger).

## Data availability

All data described are available in this manuscript and in the Supporting Information.

## Supporting information

This article contains Supporting Information.

## Conflict of interest

The authors declare that they have no conflicts of interest with the contents of this article.

## Supporting information

MS Data

Supporting Information

AtPCO4 6S7E Peptidomimetic 10.pdb

AtPCO4 6S7E Peptidomimetic 10.pse

AtPCO4 6S7E RAP2.12.pdb

AtPCO4 6S7E RAP2.12.pse
