# Supplementary material for: Evidence of an activity-enhancing conformational shift in Arabidopsis thaliana plant cysteine oxidase 4 induced by binding of substrate or substrate-mimics
Source: J Biol Chem. 2025 Sep 29;301(11):110770. doi: 10.1016/j.jbc.2025.110770 (PMC12630348; doi:10.1016/j.jbc.2025.110770)
Supplement: Supporting Information [file mmc2.docx]

**Supporting Information**

**Evidence of an activity-enhancing conformational shift in *Arabidopsis thaliana* Plant Cysteine Oxidase 4 induced by binding of substrate or substrate-mimics**

R. Latter^a^, J. C. J Hintzen^b,c^, L. M. N. Shah^a,d^, D. M. Gunawardana^a^, R. M. Sher^a^, M. D. White,^e^ J. Mecinović^b^, J. L. P. Benesch^a,d^, E. Flashman^f*^

*^a^* Department of Chemistry, University of Oxford, 12 Mansfield Road, OX1 3TA, U.K.

*^b^* Department of Physics, Chemistry and Pharmacy, University of Southern Denmark, Campusvej 55, 5230 Odense, Denmark.

^c^ Present address: Department of Biochemistry and Biophysics, Perelman School of Medicine, University of Pennsylvania, Philadelphia, PA, 19104, USA.

^d^Kavli Institute for Nanoscience Discovery, University of Oxford, South Parks Road, OX1 3QU, U.K.

^e^ School of Chemistry, University of Sydney, NSW, Australia.

^f^ Department of Biology, University of Oxford, South Parks Road, OX1 3RB, U.K.

**Corresponding author:* emily.flashman@biology.ox.ac.uk

**Includes supporting:**

**Characterisation of peptidomimetics**

**Table S1** Identity and MS characterisation of the peptidomimetic series.

**Figure S1** Analytical HPLC after RP-HPLC purification and MALDI-TOF MS spectra of peptidomimetic **1**

**Figure S2** Analytical HPLC after RP-HPLC purification and MALDI-TOF MS spectra of peptidomimetic **2**

**Figure S3** Analytical HPLC after RP-HPLC purification and MALDI-TOF MS spectra of peptidomimetic **3**

**Figure S4** Analytical HPLC after RP-HPLC purification and MALDI-TOF MS spectra of peptidomimetic **4**

**Figure S5** Analytical HPLC after RP-HPLC purification and MALDI-TOF MS spectra of peptidomimetic **5**

**Figure S6** Analytical HPLC after RP-HPLC purification and MALDI-TOF MS spectra of peptidomimetic **6**

**Figure S7** Analytical HPLC after RP-HPLC purification and MALDI-TOF MS spectra of peptidomimetic **7**

**Figure S8** Analytical HPLC after RP-HPLC purification and MALDI-TOF MS spectra of peptidomimetic **8**

**Figure S9** Analytical HPLC after RP-HPLC purification and MALDI-TOF MS spectra of peptidomimetic **9**

**Figure S10** Analytical HPLC after RP-HPLC purification and MALDI-TOF MS spectra of peptidomimetic **10**

**Figure S11** Analytical HPLC after RP-HPLC purification and MALDI-TOF MS spectra of peptidomimetic **11**

**Figure S12** Analytical HPLC after RP-HPLC purification and MALDI-TOF MS spectra of peptidomimetic **12**

**Figure S13** Analytical HPLC after RP-HPLC purification and MALDI-TOF MS spectra of peptidomimetic **13**

**Figure S14** Mass spectra of peptidomimetics prior to and following a 20 min incubation with AtPCO4

**Supporting *in vitro* assays and experimental set up**

**Figure S15** Investigating the potential formation of a Cys190-Tyr192 activity-enhancing crosslink.

**Figure S16** AtPCO4 activity towards RAP2.12_2—15_ or RAP2.12_2—17_ in the presence and absence of peptidomimetic **10**

**Figure S17** AtPCO4 ADO loop variant and WT AtPCO4 activity towards RAP2.12_2—17_ in the presence and absence of peptidomimetic **9**

**Figure S18** AC_50_ graph for peptidomimetic **9**

**Supporting HDX-MS results**

**Figure S19** Woods plots of statistical significance testing for peptide fragments generated by HDX-MS experiments

**Characterisation of Peptidomimetics**

Table S1 and Figures S1-13

| **Table S1** Identity and MS characterisation of the peptidomimetic series. | | | | | |
| --- | --- | --- | --- | --- | --- |
| **Peptidomimetic** | | **Sequence** | **Formula** | **m/z Calculated** | **m/z Found** |
| **1** | d-Cys | d-CysGGAIISDFIPPPR | C_65_H_104_N_18_O_17_S | 1441.8 | 1442.5 |
| **2** | hCys | hCysCysGGAIISDFIPPR | C_66_H_106_N_18_O_17_S | 1455.8 | 1457.1 |
| **3** | Pen | PenGGAIISDFIPPPR | C_67_H_108_N_18_O_17_S | 1469.8 | 1470.1 |
| **4** | SmeC | SMeCysGGAIISDFIPPPR | C_66_H_106_N_18_O_17_S | 1455.8 | 1457.1 |
| **5** | Thz | ThzGGAIISDFIPPPR | C_66_H_104_N_18_O_17_S | 1453.8 | 1454.1 |
| **6** | TM3C | TM3CGGAIISDFIPPPR | C_67_H_106_N_18_O_17_S | 1467.8 | 1468.3 |
| **7** | 3MePro | 3MeProGGAIISDFIPPR | C_65_H_103_N_17_O_17_S | 1426.7 | 1427.0 |
| **8** | N-Me-Cys | Nme-CysGGAIISDFIPPR | C_66_H_106_N_18_O_17_S | 1455.8 | 1456.0 |
| **9** | Abu | AbuCGGAIISDFIPPPR | C_66_H_106_N_18_O_17_ | 1423.8 | 1425.1 |
| **10** | Ser | SGGAIISDFIPPPR | C_65_H_104_N_18_O_18_ | 1425.8 | 1426.2 |
| **11** | hSer | hSerGGAIISDFIPPPR | C_66_H_106_N_18_O_18_ | 1438.8 | 1440.2 |
| **12** | AlG | AlGGGAIISDFIPPPR | C_67_H_106_N_18_O_17_ | 1435.8 | 1436.2 |
| **13** | Pra | PraCGGAIISDFIPPPR | C_67_H_104_N_18_O_17_ | 1433.8 | 1434.2 |

**A**

**
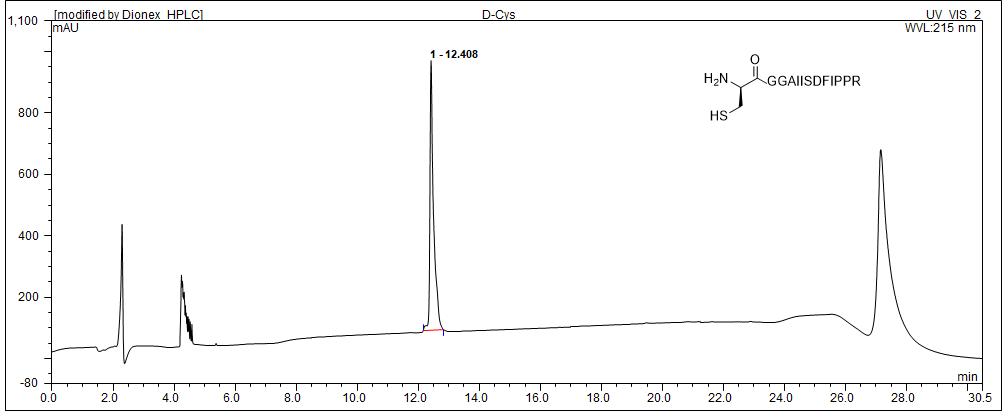
**

**B**


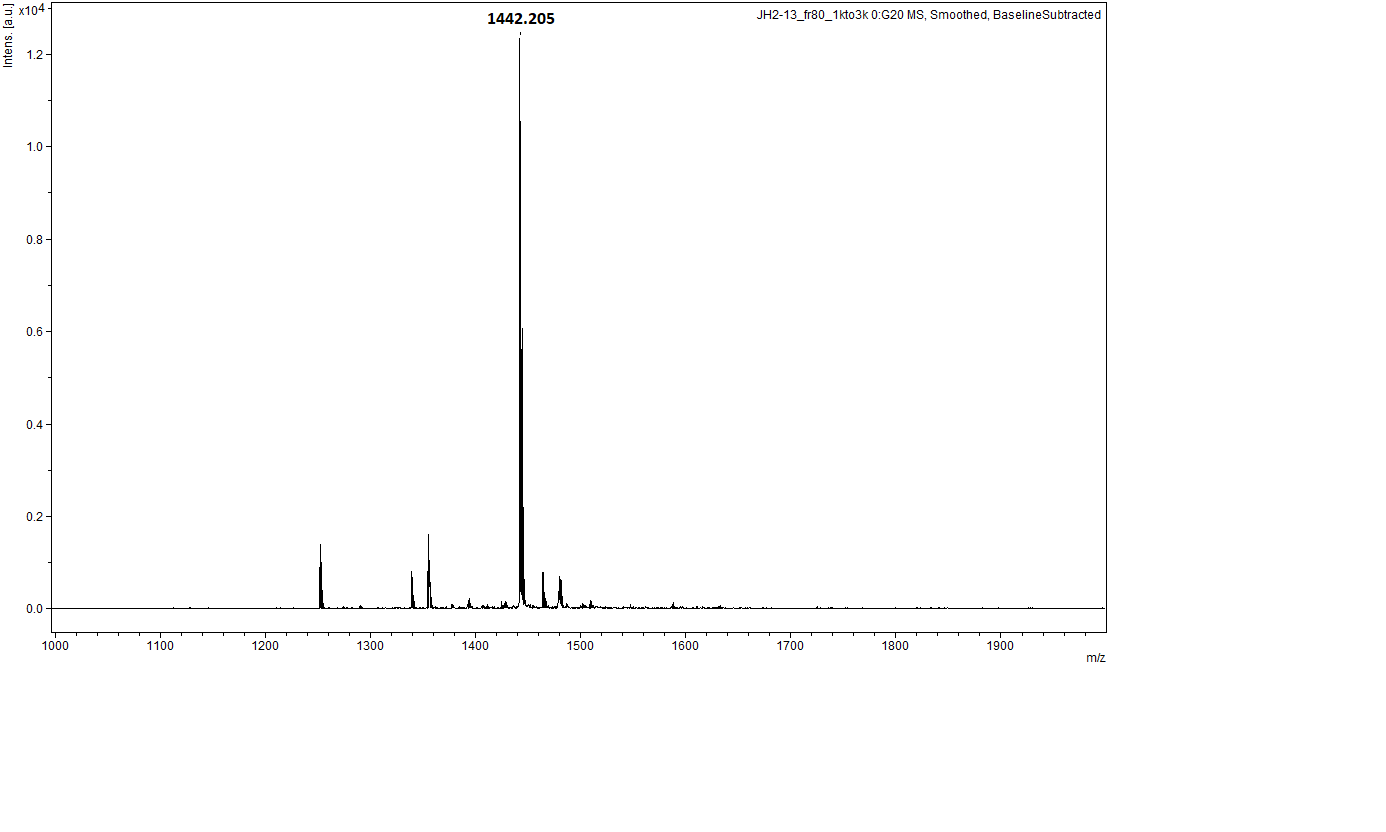


**Figure S1 A** Analytical HPLC of the d-Cysteine containing peptidomimetic **1** after RP-HPLC purification. **B** MALDI-TOF MS spectra of the purified d-Cysteine containing peptidomimetic **1**.

**A**
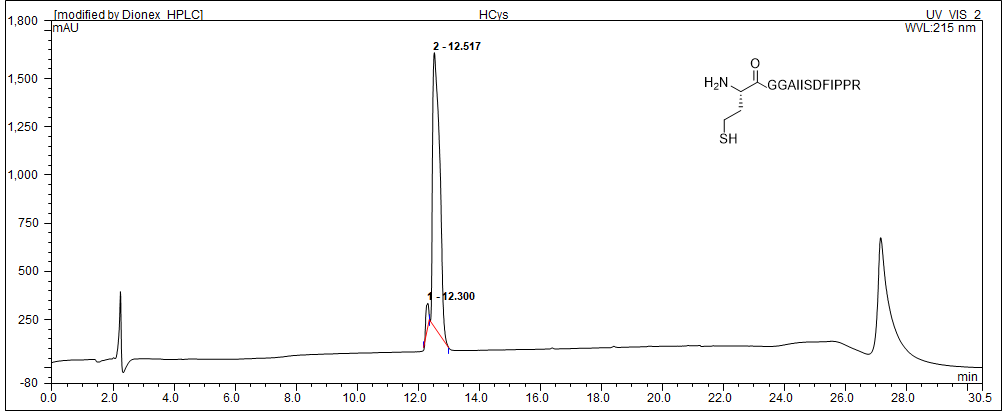


**B**


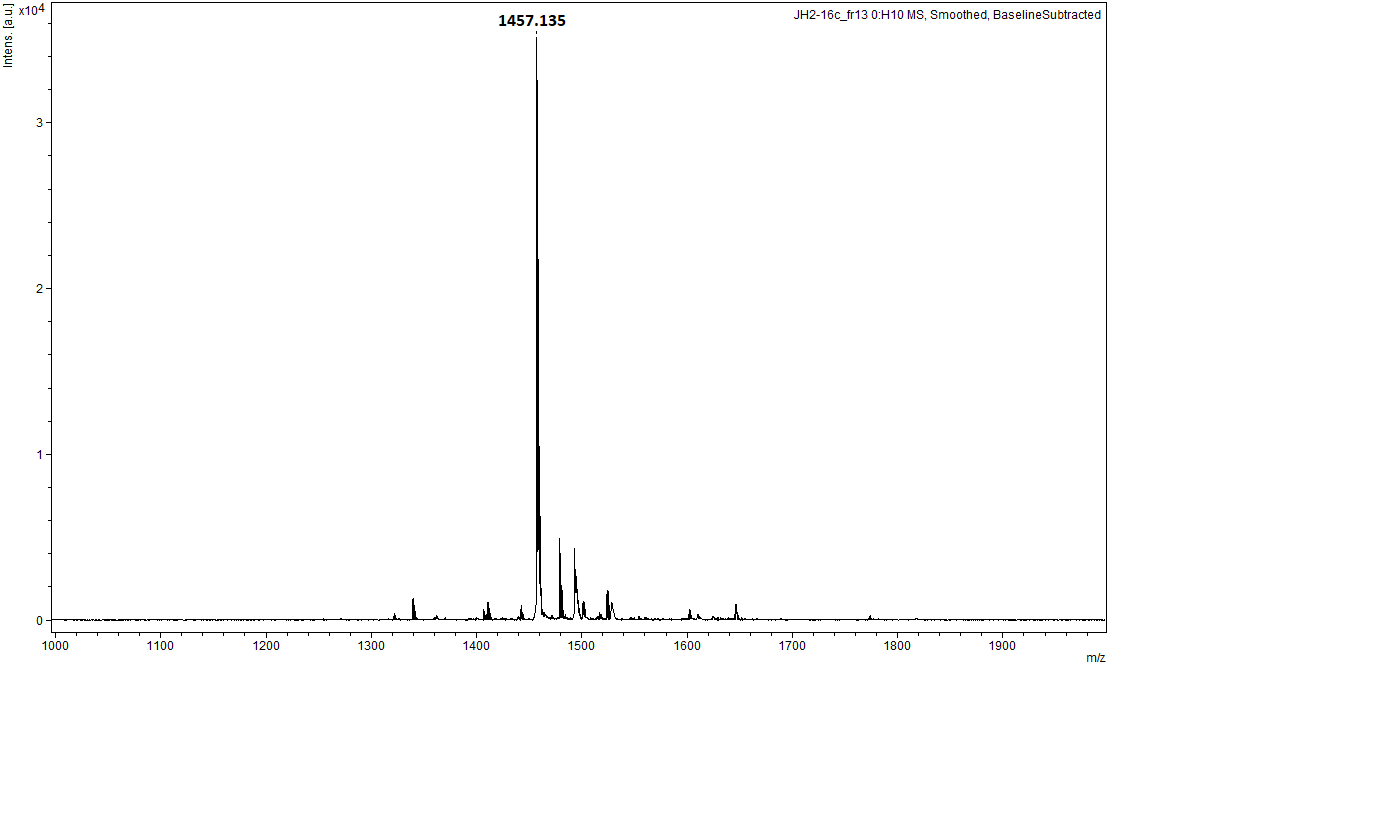


**Figure S2 A** Analytical HPLC of the homocysteine containing peptidomimetic **2** after RP-HPLC purification. **B** MALDI-TOF MS spectra of the purified homocysteine containing peptidomimetic **2**.

**A**
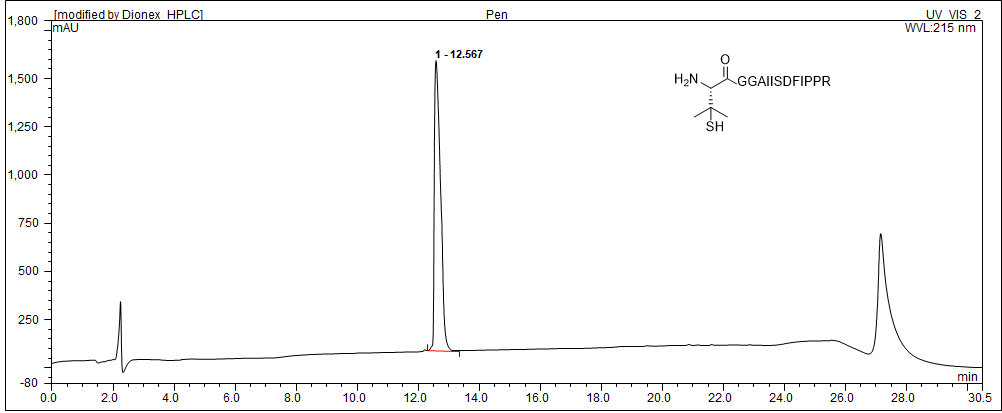


**B**


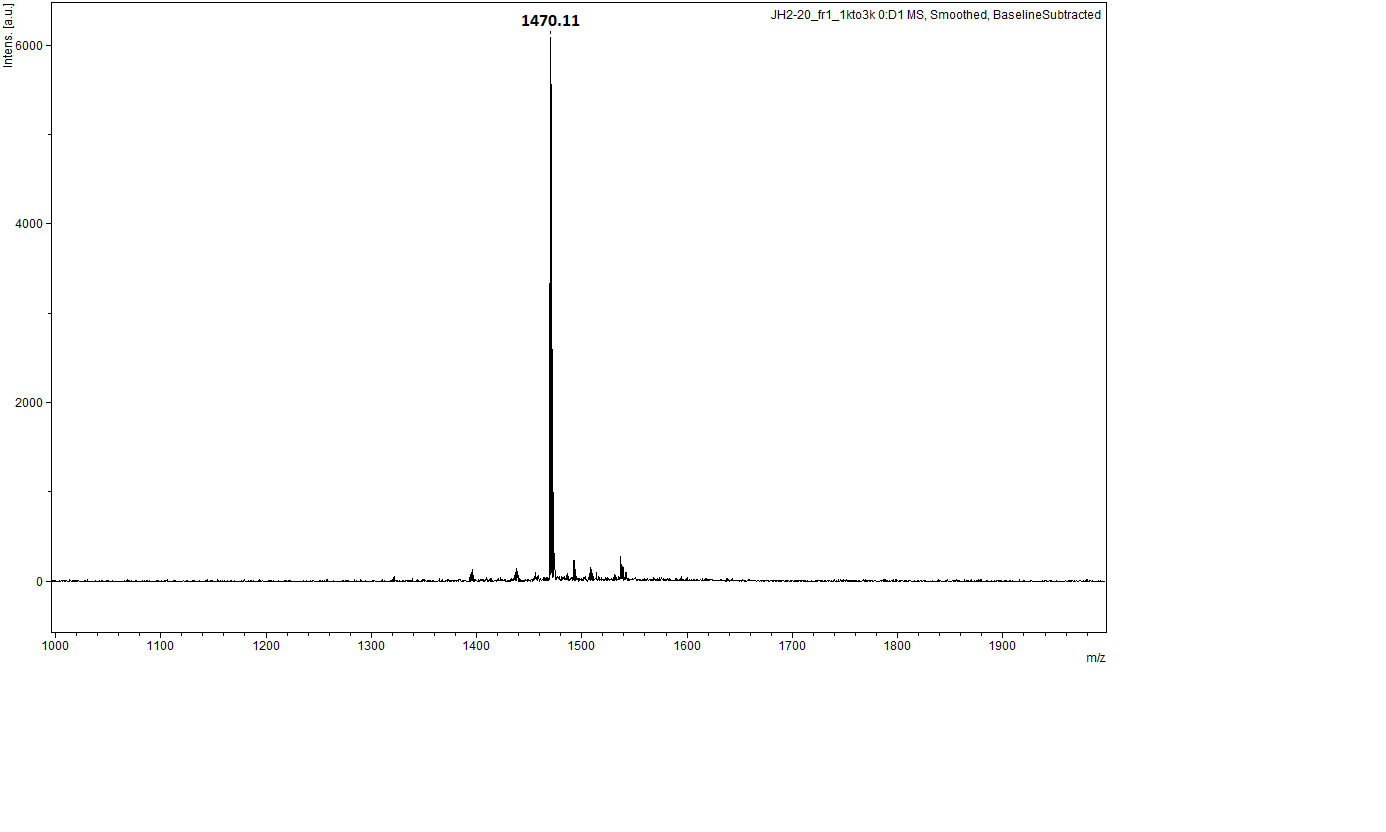


**Figure S3 A** Analytical HPLC of the penicillamine containing peptidomimetic **3** after RP-HPLC purification. **B** MALDI-TOF MS spectra of the purified penicillamine containing peptidomimetic **3**.

**A**
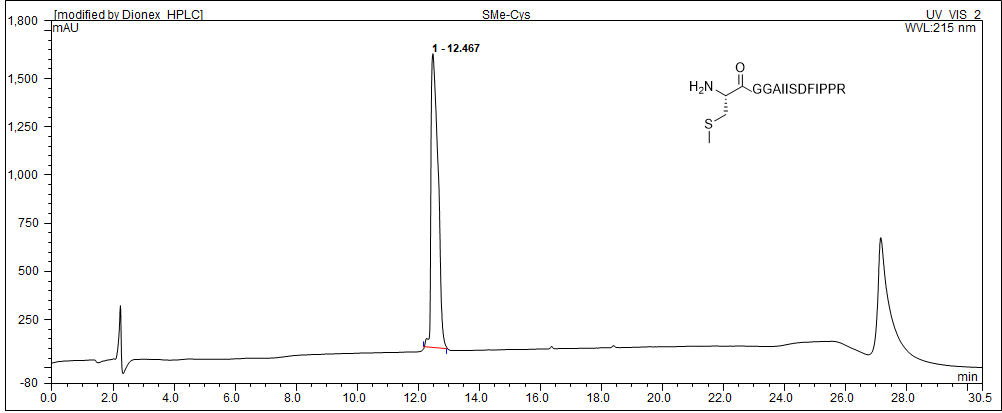


**B**


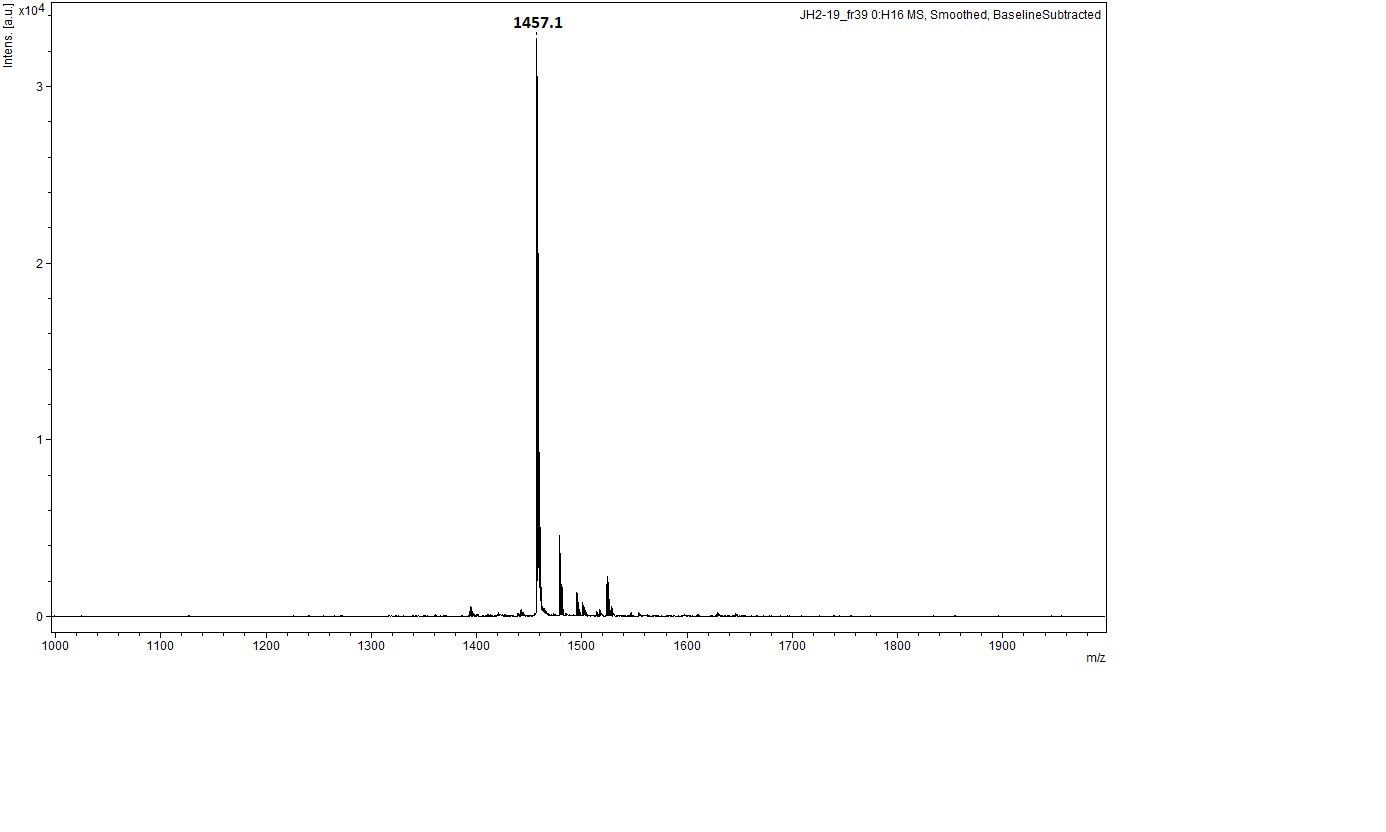


**Figure S4 A** Analytical HPLC of the Sme-cysteine containing peptidomimetic **4** after RP-HPLC purification. **B** MALDI-TOF MS spectra of the purified Sme-cysteine containing peptidomimetic **4**.

**A**
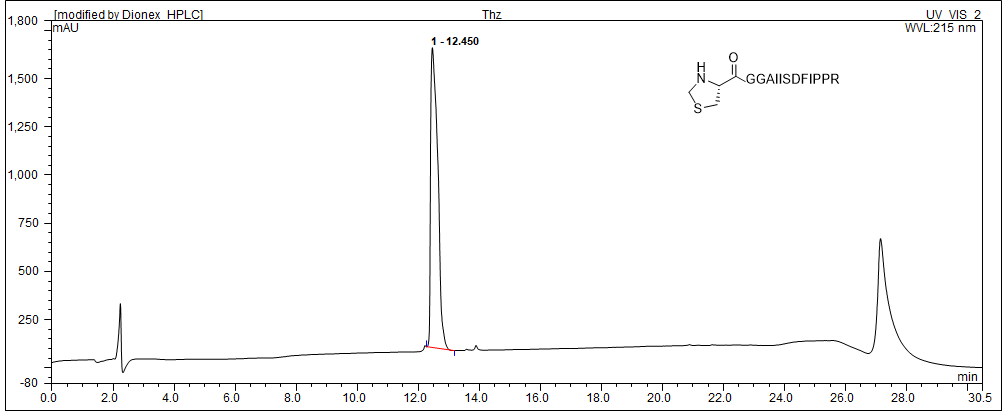


**B**


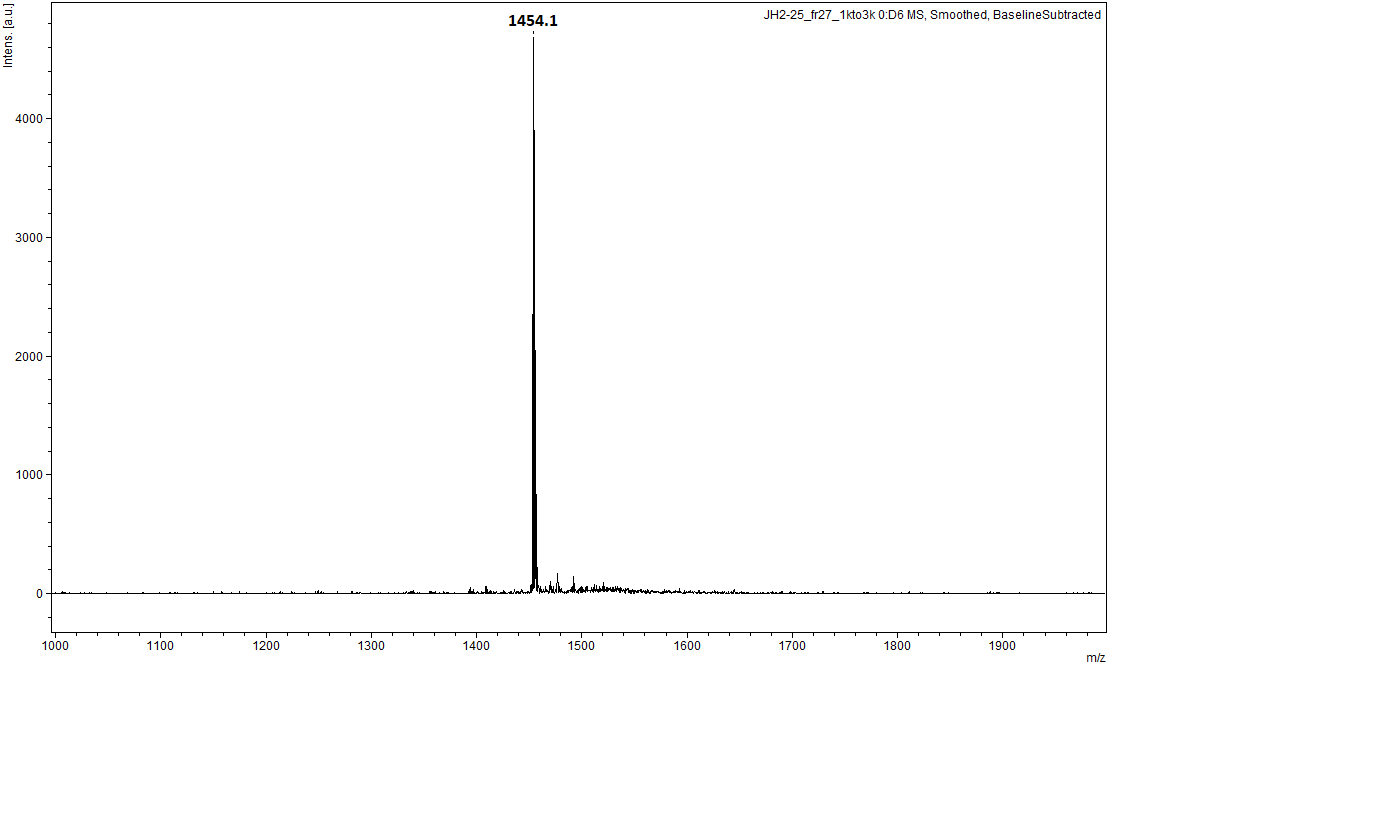


**Figure S5 A** Analytical HPLC of the thiazolidine containing peptidomimetic **5** after RP-HPLC purification. **B** MALDI-TOF MS spectra of the purified thiazolidine containing peptidomimetic **5**.

**A**
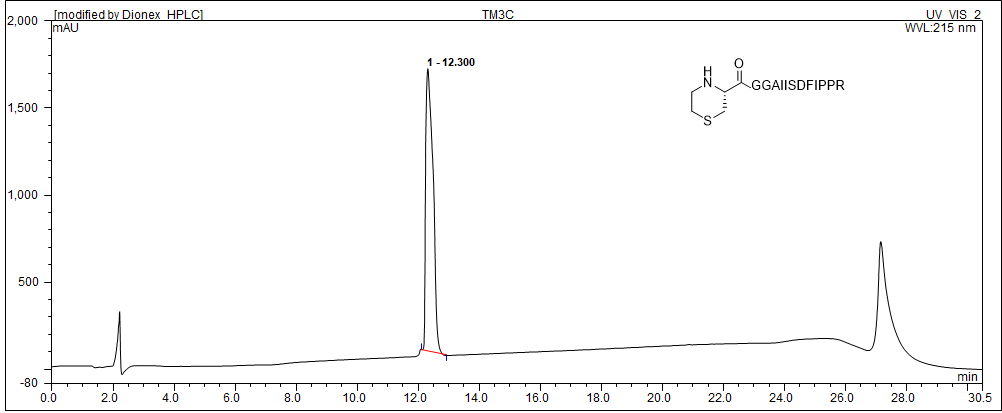


**B**


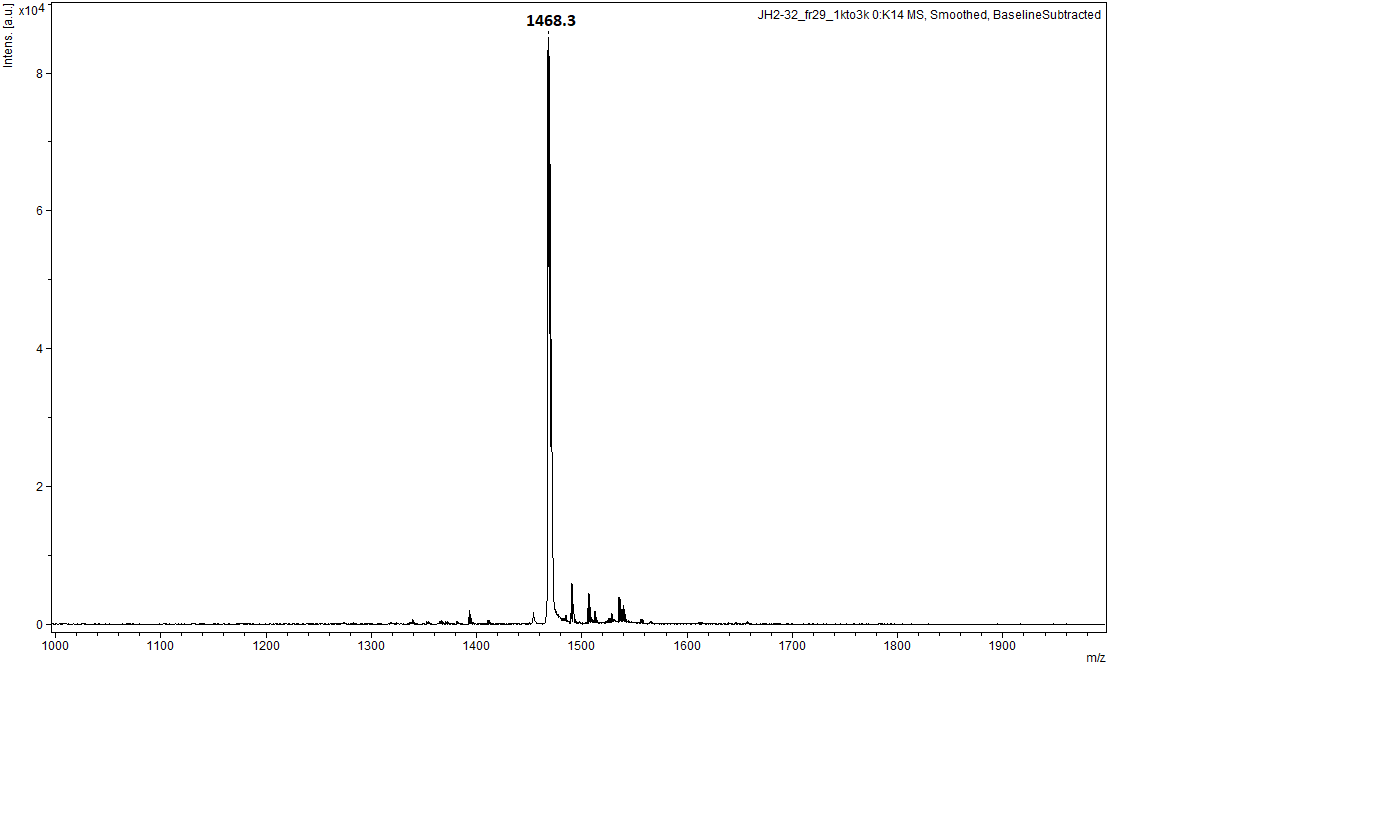


**Figure S6 A** Analytical HPLC of the thiomorpholine containing peptidomimetic **6** after RP-HPLC purification. **B** MALDI-TOF MS spectra of the purified thiomorpholine containing peptidomimetic **6**.

**A**

**
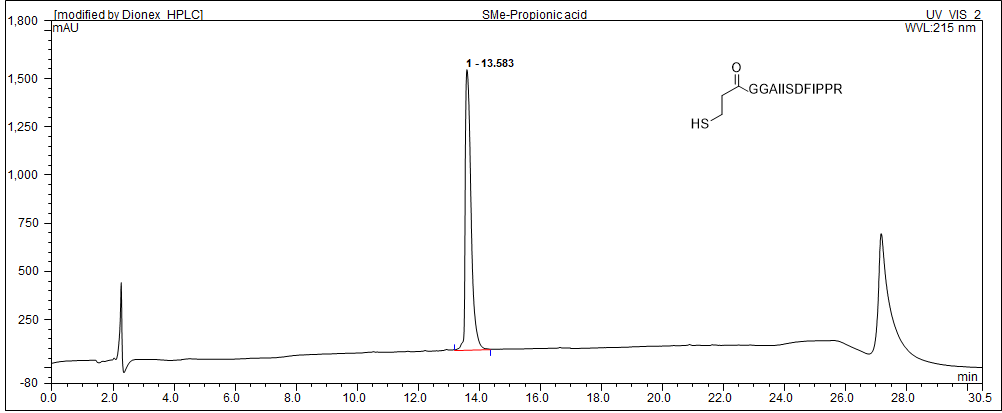
**

**B**


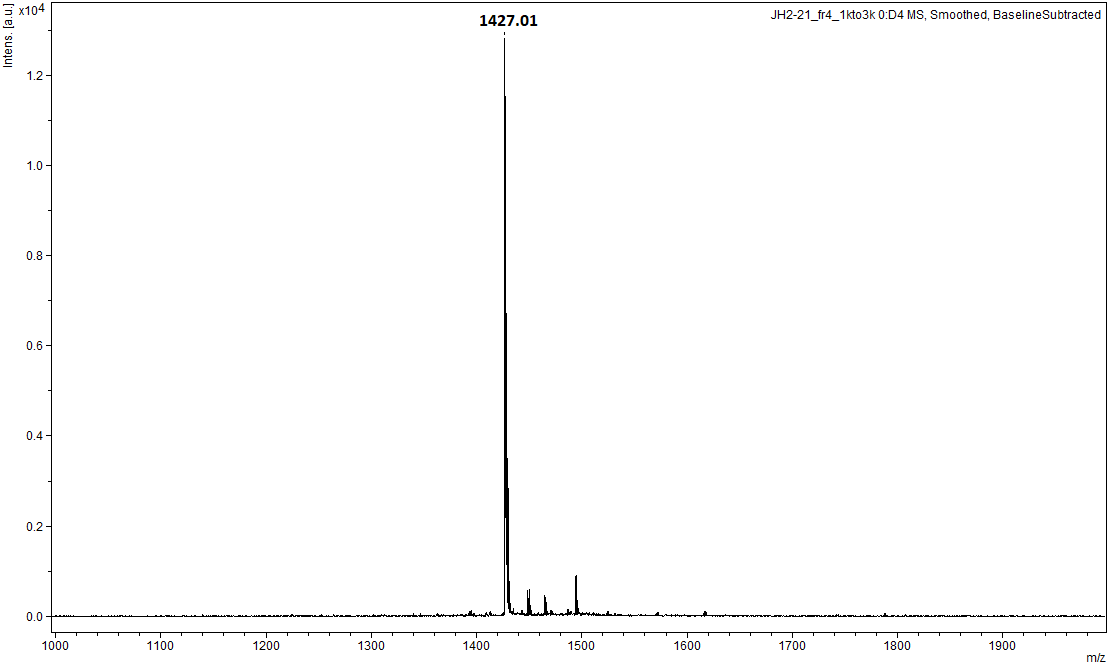


**Figure S7. A** Analytical HPLC of the 3-mercapto propionic acid containing peptidomimetic **7** after RP-HPLC purification. **B** MALDI-TOF MS spectra of the purified 3-mercapto propionic acid containing peptidomimetic **7**.

**A**
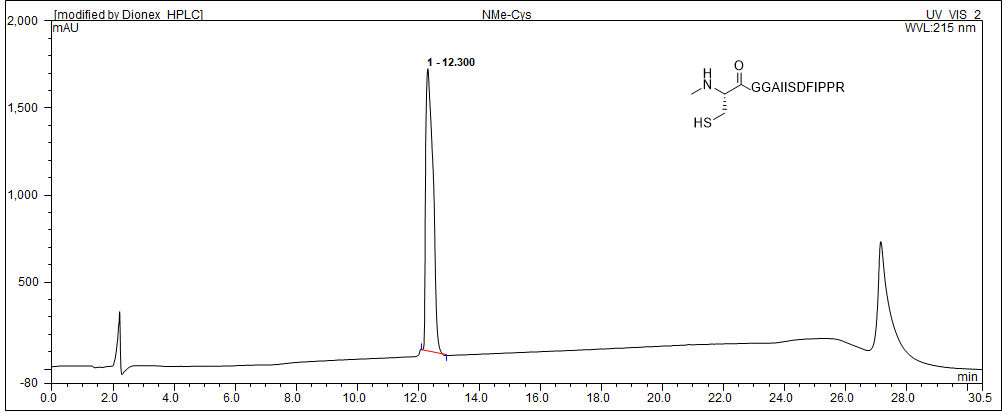


**B**


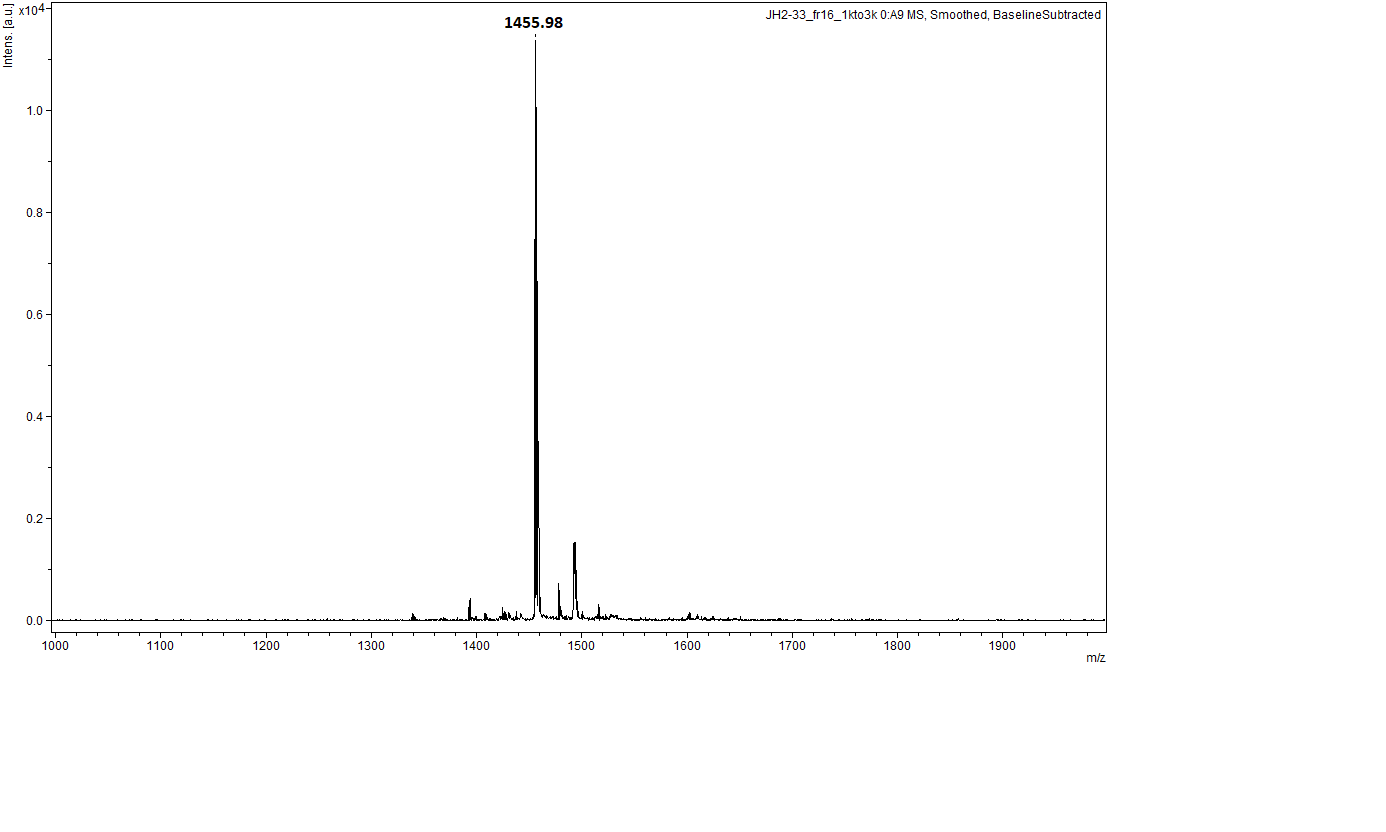


**Figure S8 A** Analytical HPLC of the N-Me-cysteine containing peptidomimetic **8** after RP-HPLC purification. **B** MALDI-TOF MS spectra of the purified N-Me-cysteine containing peptidomimetic **8**.

**A**
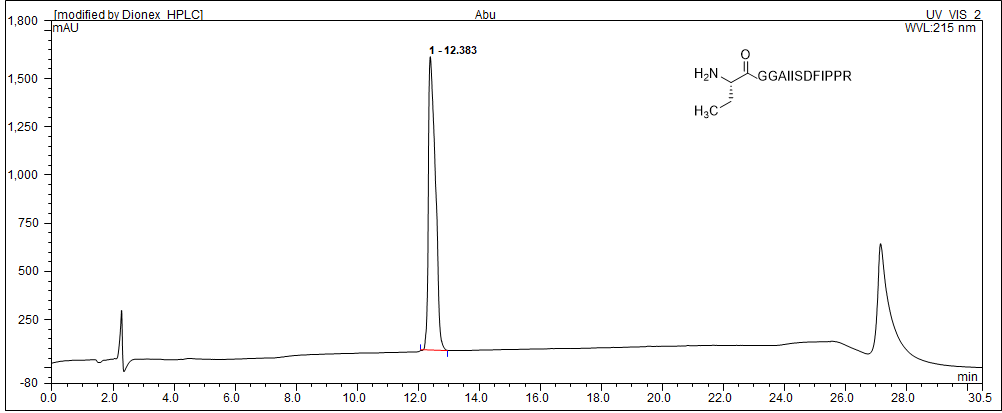


**B**


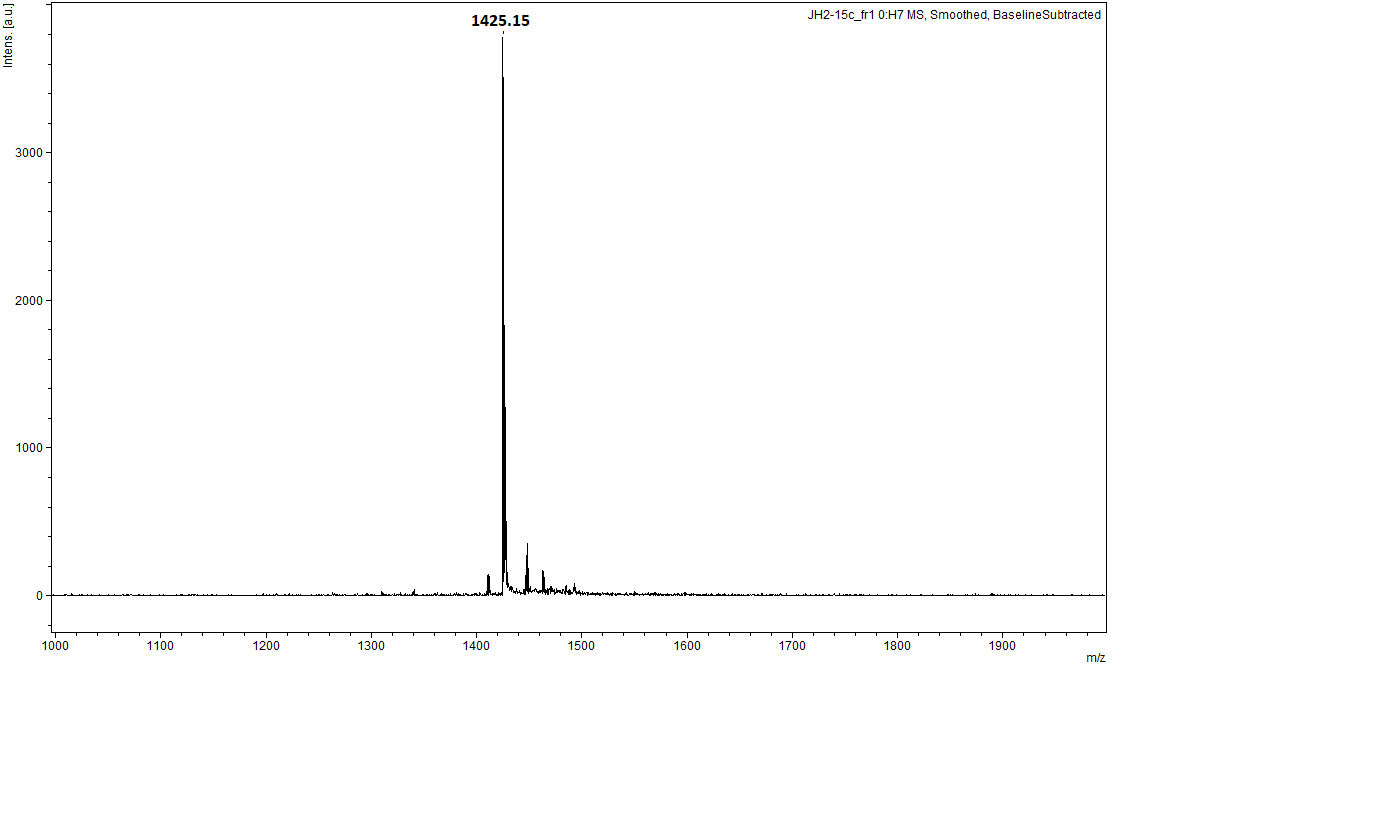


**Figure S9 A** Analytical HPLC of the aminobutyric acid (hAla) containing peptidomimetic **9** after RP-HPLC purification. **B** MALDI-TOF MS spectra of the purified aminobutyric acid (hAla) containing peptidomimetic **9**.

**A**
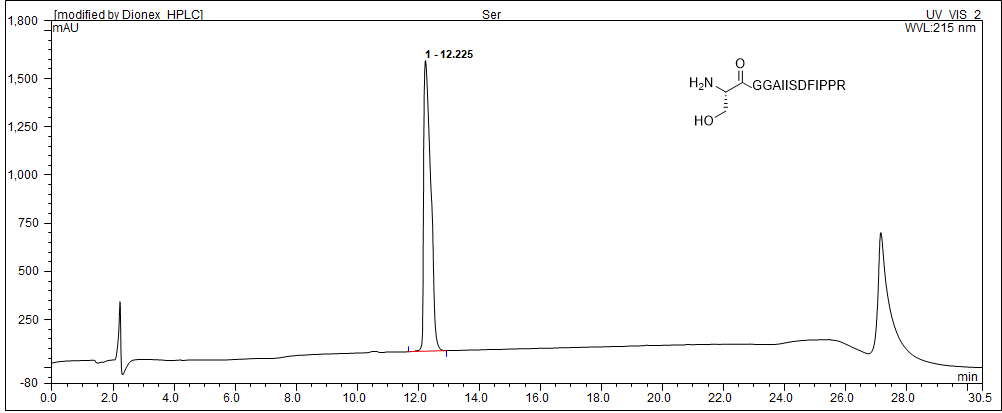


**B**


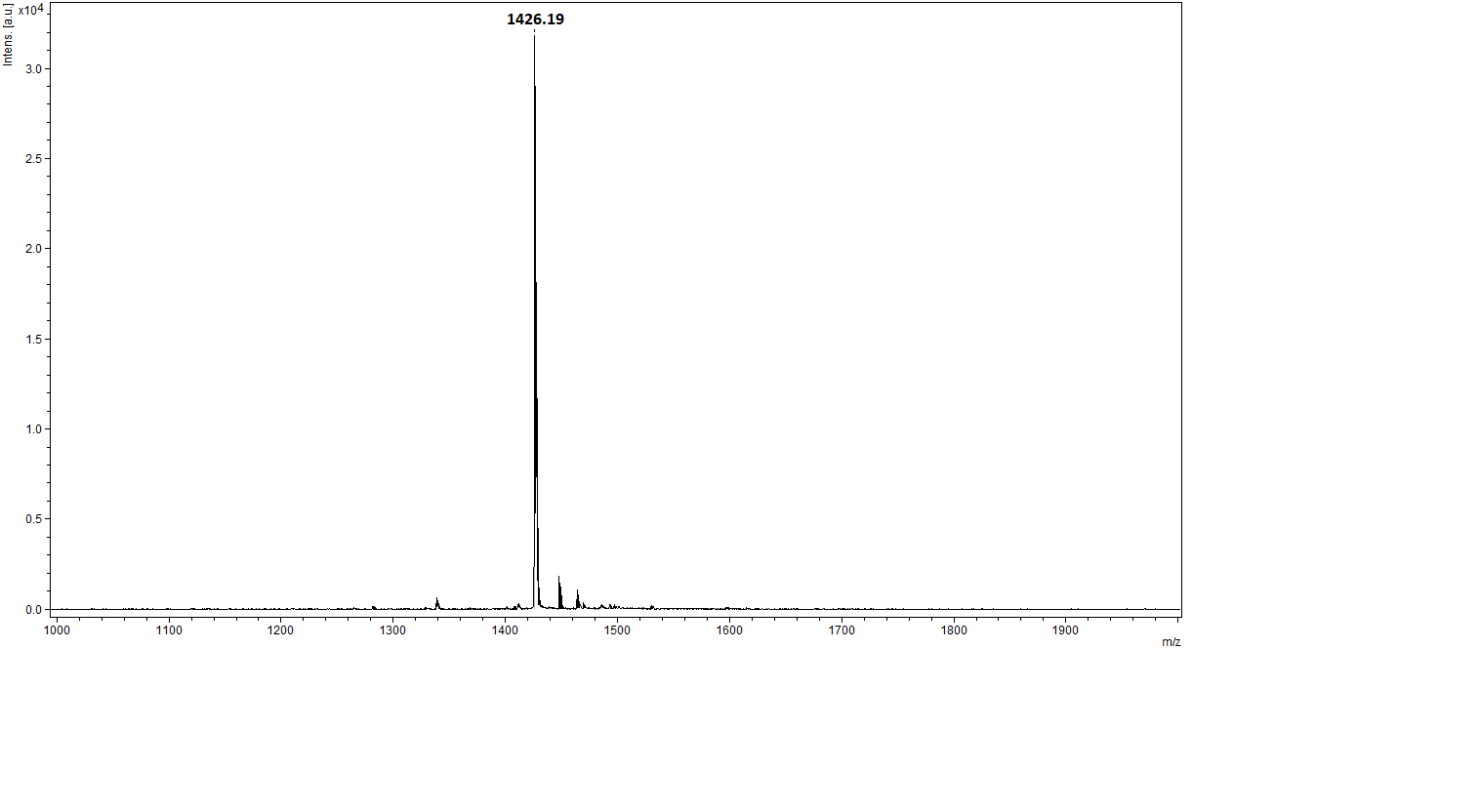


**Figure S10 A** Analytical HPLC of the serine containing peptidomimetic **10** after RP-HPLC purification. **B** MALDI-TOF MS spectra of the purified serine containing peptidomimetic **10**.

**A**
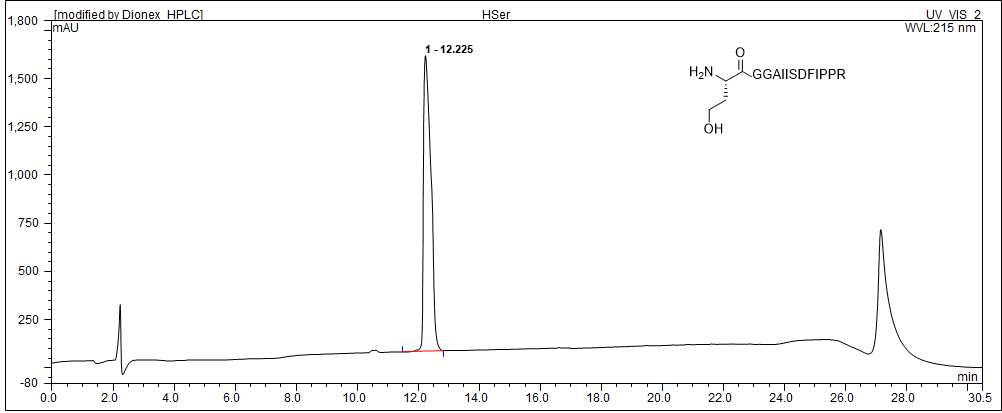


**B**


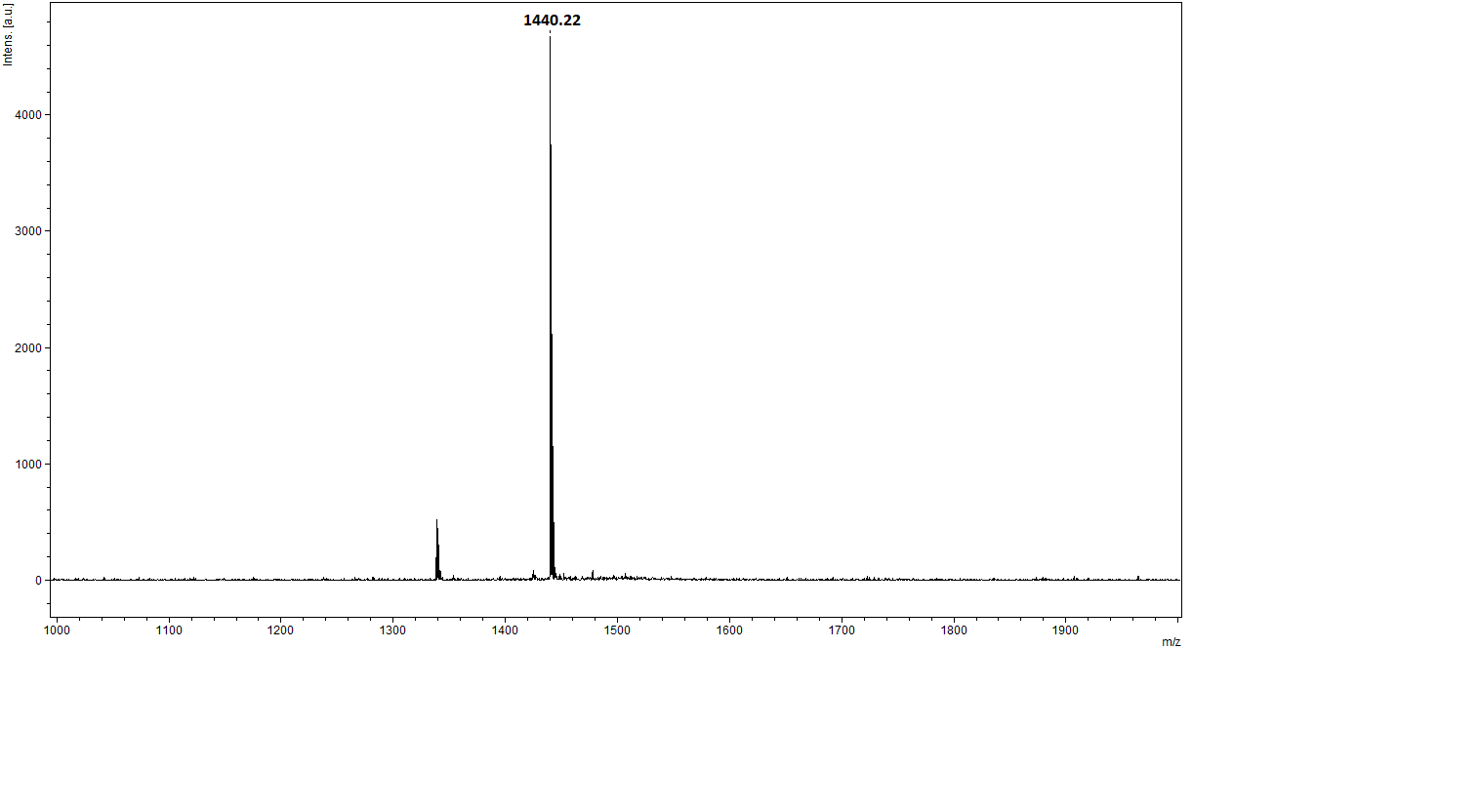


**Figure S11 A** Analytical HPLC of the homoserine containing peptidomimetic **11** after RP-HPLC purification. **B** MALDI-TOF MS spectra of the purified homoserine containing peptidomimetic **11**.

**A**
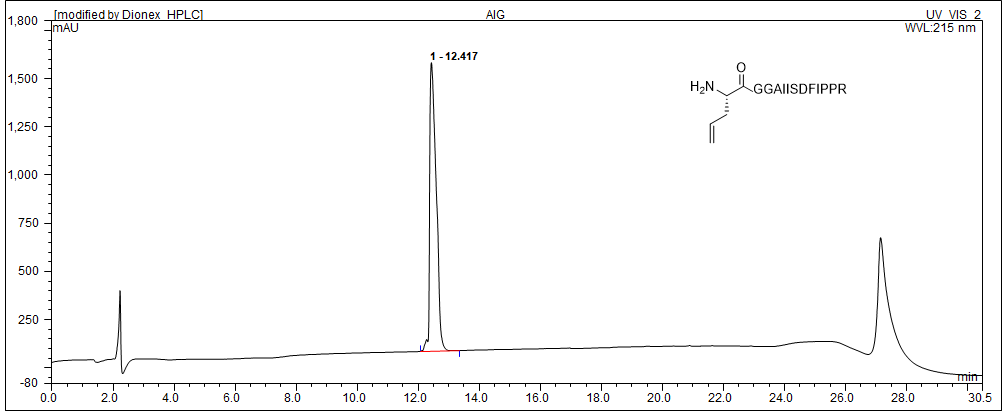


**B**


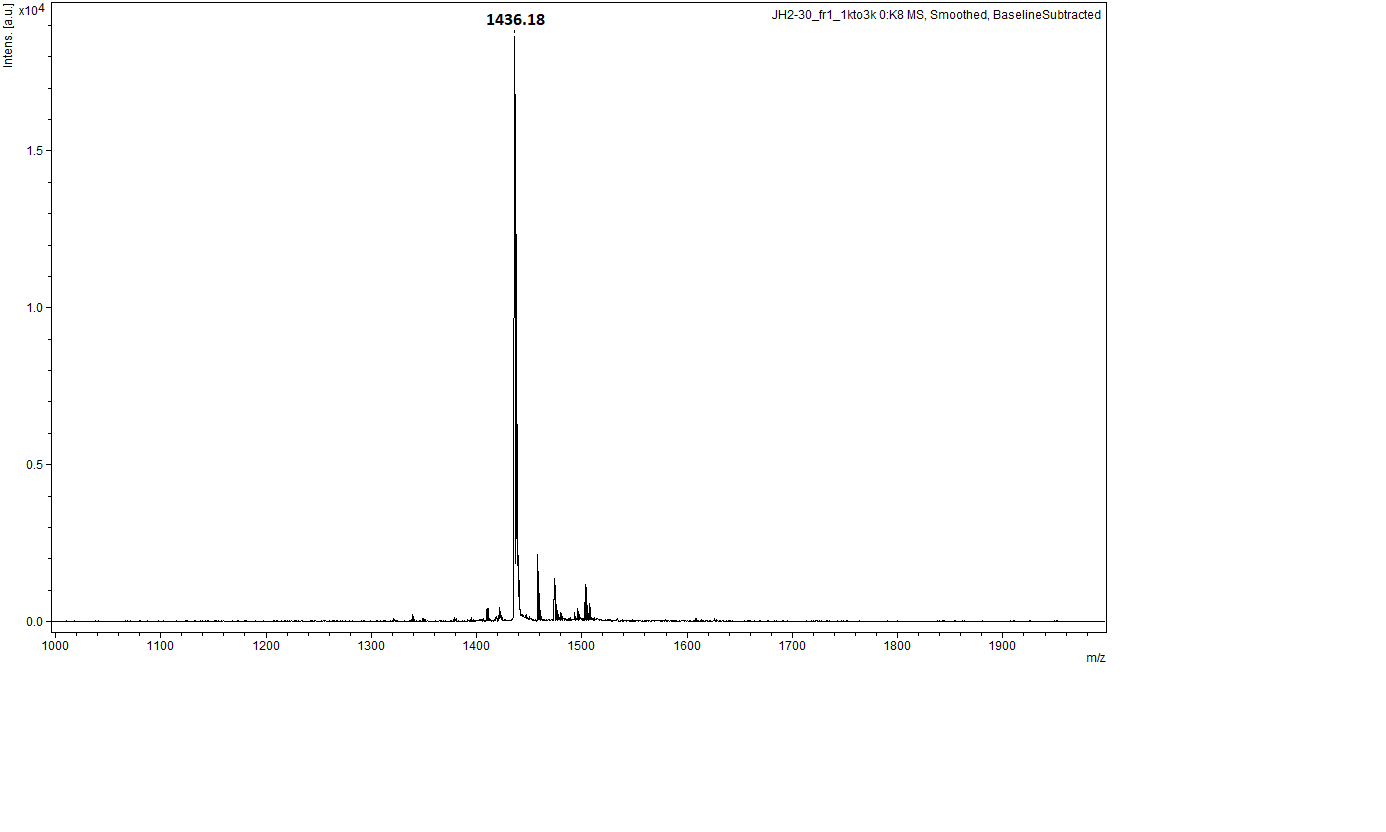


**Figure S12 A** Analytical HPLC of the allylglycine containing peptidomimetic **12** after RP-HPLC purification. **B** MALDI-TOF MS spectra of the purified allyglycine containing peptidomimetic **12**.

**A**
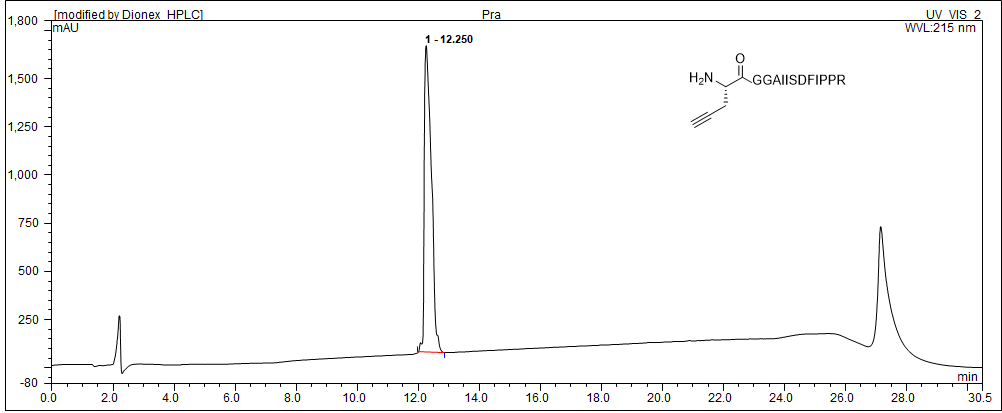


**B**


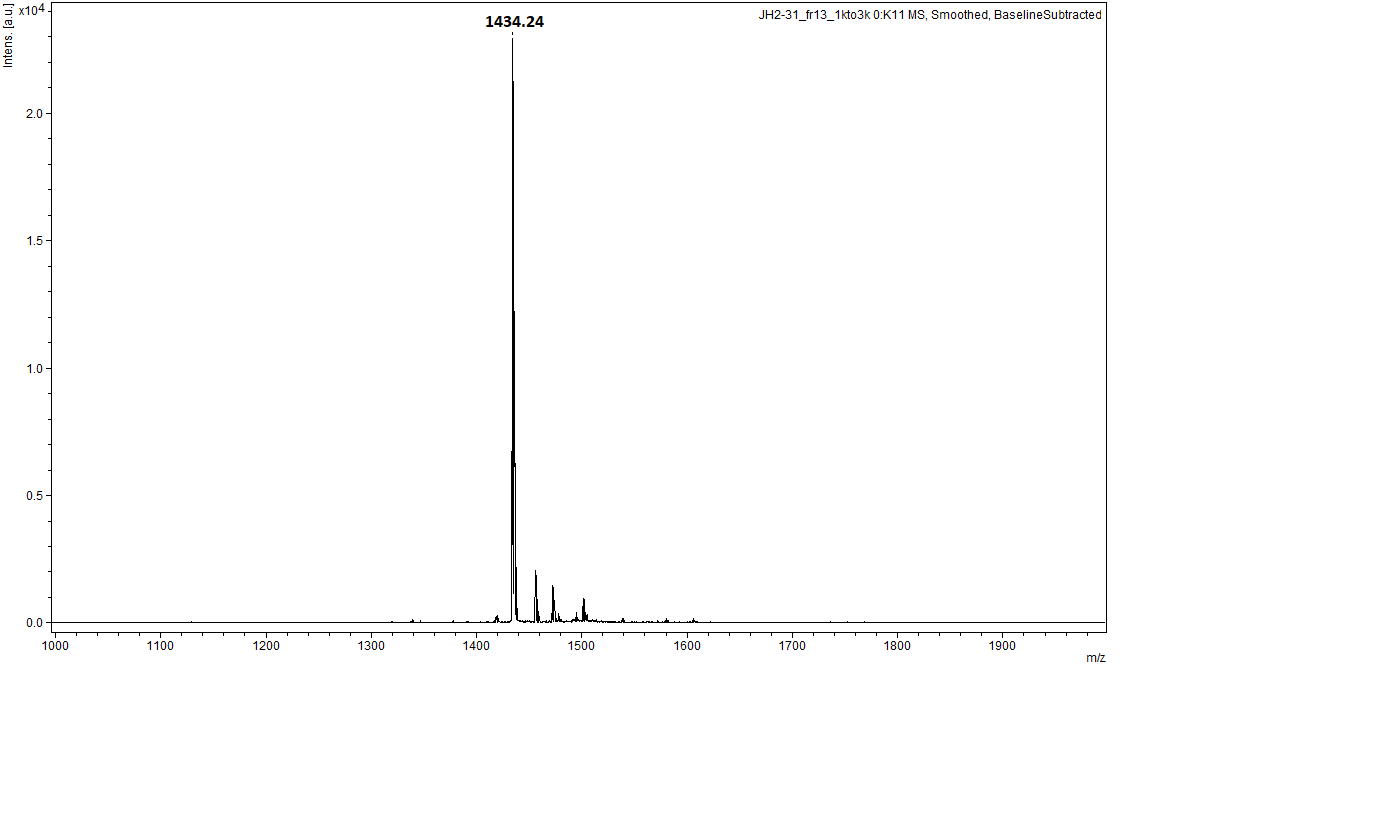


**Figure S13 A** Analytical HPLC of the propargylglycine containing peptidomimetic **13** after RP-HPLC purification. **B** MALDI-TOF MS spectra of the purified propargylglycine containing peptidomimetic **13**.

**
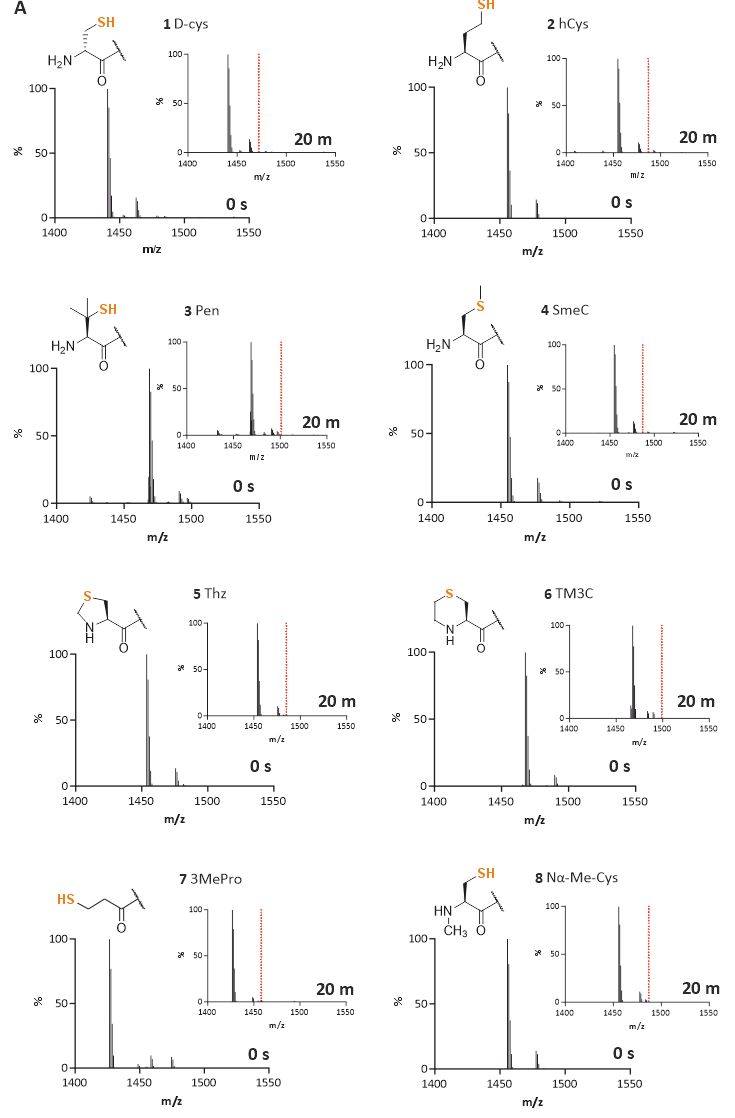
**

**
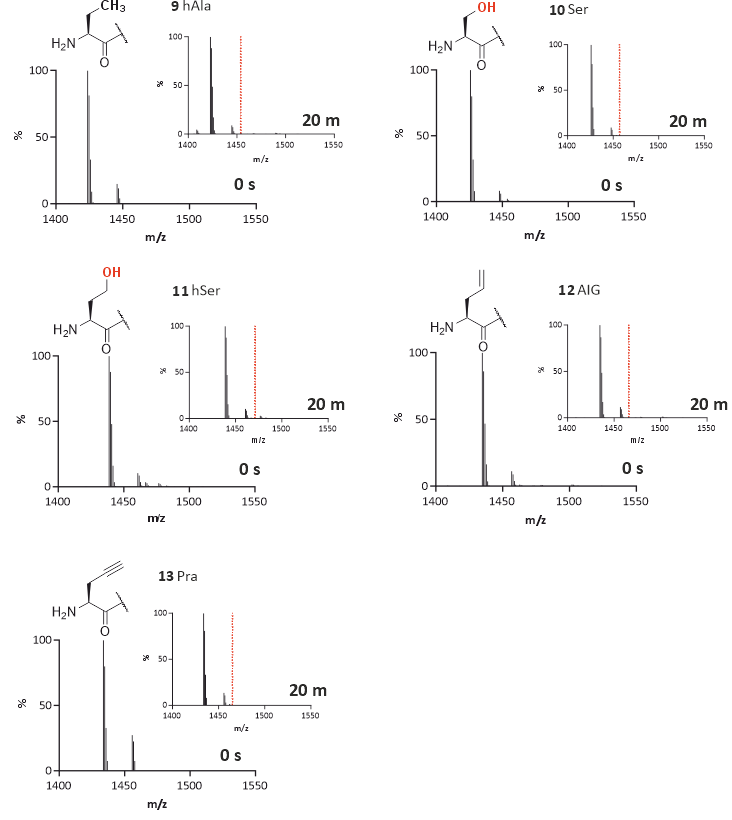
**

**Figure S14** **Mass spectra of peptidomimetics before and following a 20 min incubation with AtPCO4. A** Structure of the N-terminal Cys-analogue for each of the peptidomimetics are shown along with vertical red dashed lines indicating where +32 Da peaks would be expected if AtPCO4 catalysed the addition of molecular oxygen to the peptidomimetics. Standard conditions: 0.1 μM AtPCO4, 200 μM peptidomimetic, 5 mM TCEP, 20 μM FeSO_4_, 1 mM ascorbate, 25 °C.

**Supporting *in vitro* assays**

**
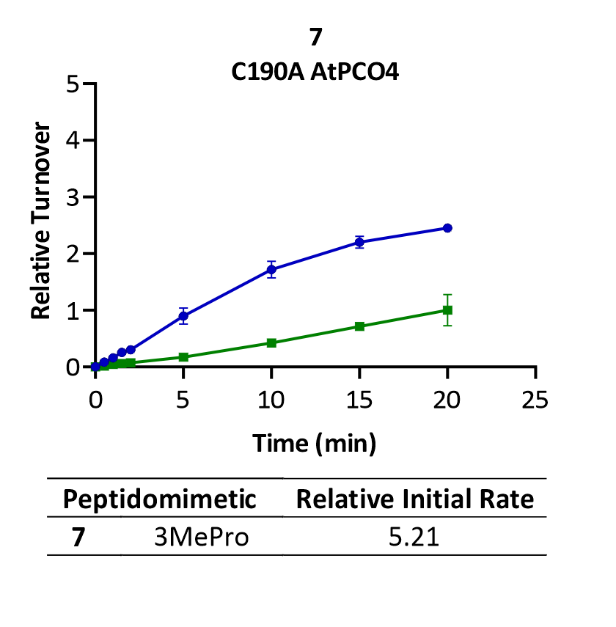
**

**Figure S15 Investigating the potential formation of a Cys190-Tyr192 activity-enhancing crosslink.** *In vitro* time course activity assays showing relative turnover of 20 μM RAP2.12_2—17_ by 0.1 μM C190A AtPCO4 variant following preincubation in the absence (green) and presence (blue) of 200 μM peptidomimetic **7**. Standard conditions: 5 mM TCEP, 20 μM FeSO_4_, 1 mM ascorbate, 25 °C. Turnover is calculated as μmol RAP2.12_2—17_ oxidised per μg of AtPCO4, values are normalised to RAP2.12_2—17_ turnover after 20 mins, in the absence of peptidomimetic (=1). Error bars display S.D (n=3). Relative rate of each reaction is displayed in the table and is calculated from the slope of reaction progress between 0−5 min in the presence of peptidomimetic **7**, normalised to the corresponding control condition without peptidomimetic (=1).


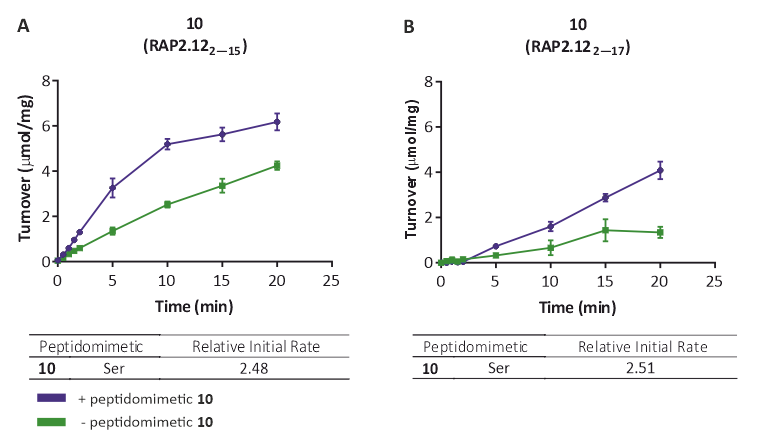


**Figure S16 AtPCO4 activity towards (A) RAP2.12_2—15_ or (B) RAP2.12_2—17_ in the presence and absence of peptidomimetic 10.** *In vitro* time course activity assays showing activity of 0.1 μM AtPCO4 towards 20 μM RAP2.12_2—15/17_ in the absence (green) and presence (blue) of 200 μM peptidomimetics **10**. Standard conditions: 5 mM TCEP, 20 μM FeSO_4_, 1 mM ascorbate, 25 °C. Turnover calculated as μmol RAP2.12_2—15/17_ oxidised per μg of AtPCO4 (data shown is not normalised). Error bars display S.D (n=3). Table below each graph shows the relative initial rates of RAP2.12_2—15/17_ turnover, calculated from the rate of reaction between 0-5 min in the presence of peptidomimetic **10**, normalised to the corresponding control condition without peptidomimetic **10** (=1). Within each pair the slopes used to calculate relative rates from 0-5 min, differ significantly (determined using Prism 10.3.1).


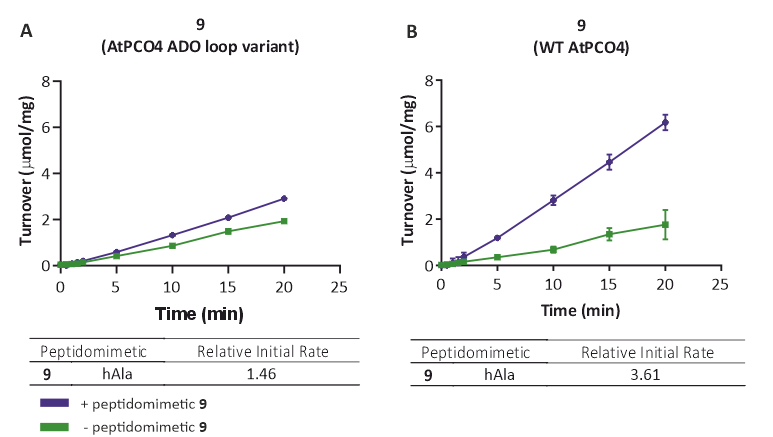


**Figure S17 (A) AtPCO4 ADO loop variant and (B) WT AtPCO4 activity towards RAP2.12_2—17_ in the presence and absence of peptidomimetic 9.** *In vitro* time course activity assays showing activity of 0.1 μM AtPCO4 towards 20 μM RAP2.12_2—17_ in the absence (green) and presence (blue) of 200 μM peptidomimetics **9**. Standard conditions: 5 mM TCEP, 20 μM FeSO_4_, 1 mM ascorbate, 25 °C. Turnover calculated as μmol RAP2.12_2—17_ oxidised per μg of AtPCO4 (data shown is not normalised). Error bars display S.D (n=3). Table below each graph shows the relative initial rates of RAP2.12_2—17_ turnover, calculated from the rate of reaction between 0-5 min in the presence of peptidomimetic **9**, normalised to the corresponding control condition without peptidomimetic **9** (=1). Within each pair the slopes used to calculate relative rates from 0-5 min, differ significantly (determined using Prism 10.3.1).


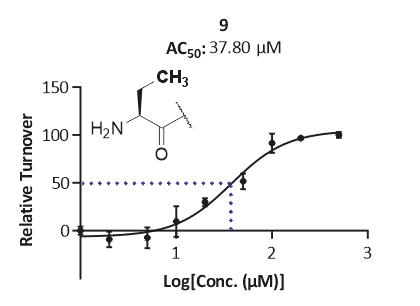


**Figure S18 AC_50_ graph for peptidomimetic 9** showing the concentration dependence of the peptidomimetic-AtPCO4 preincubation assay fitted with a variable response four-parameter curve. The blue dashed line indicates the point at which 50% maximal activity is observed, AC_50_ value 37.80 μM for peptidomimetic **9**, 95% confidence intervals 23.02 to 65.46 μM. Standard conditions: 5 mM TCEP, 20 μM FeSO_4_, 1 mM ascorbate, 25 °C. Turnover is calculated as μmol RAP2.12_2—17_ oxidised per μg of AtPCO4, the extra turnover induced by peptidomimetic **9** was calculated by subtracting the substrate-only control turnover value from that of each AtPCO4-peptidomimetic preincubation reaction. Turnover values were then normalised, the lowest value assigned to 0% and the highest to 100%. Corresponding peptidomimetic **9** concentrations were plotted as Log (μM) on the x-axis. Curve fitting in Prism 10 (GraphPad). Error bars display S.D (n=3)

**Supporting HDX-MS results**


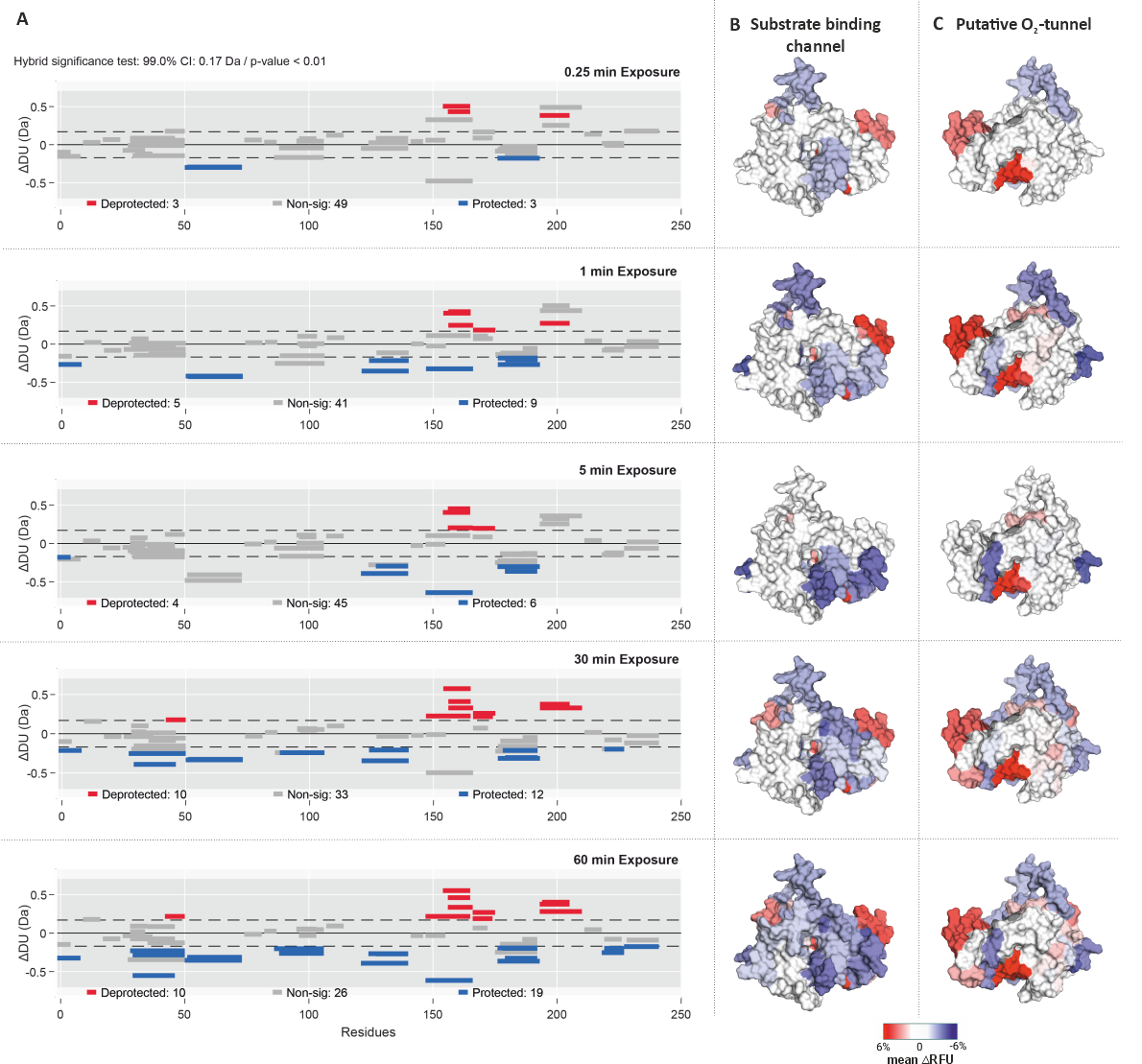


**Figure S19 Woods plots of statistical significance testing for peptide fragments generated by HDX-MS experiments. A** Peptide fragments coloured based on increased (red), decreased (blue) or no significant (grey) deuterium uptake (∆DU) of AtPCO4 upon preincubation with peptidomimetic **9** relative to the D_2_O-treated AtPCO4-only control for samples taken after 30 s, 1 min, 5 min, 30 min or 60 min of labelling. **B&C** ∆RFU values of significant and non-significant peptides are visualised using PyMOL on a surface representation of an AlphaFold 2 model of AtPCO4 displaying both the substrate binding channel (**B**) and putative O_2_ tunnel (**C**) views.
